# Effects of Dietary *Bacillus subtilis* and Bacteriophage Supplementation on Water Quality, Carcass Traits, and Muscle Growth in Magang Geese

**DOI:** 10.3390/vetsci12090861

**Published:** 2025-09-04

**Authors:** Yong Li, Yongquan Luo, Yuanhao Han, Zhiyuan Liu, Songchao Li, Xiujin Li, Zhongping Wu, Yunbo Tian, Yunmao Huang, Xumeng Zhang

**Affiliations:** College of Animal Science & Technology, Zhongkai University of Agriculture and Engineering, Guangzhou 510225, China; liyong@zhku.edu.cn (Y.L.); luoyongquan@zhku.edu.cn (Y.L.); hanyuanhao@zhku.edu.cn (Y.H.); liuzhiyuan@zhku.edu.cn (Z.L.); lisongchao@zhku.edu.cn (S.L.); lixiujin@zhku.edu.cn (X.L.); wuzhongping@zhku.edu.cn (Z.W.); tianyunbo@zhku.edu.cn (Y.T.)

**Keywords:** goose, carcass trait, skeletal muscle, *Bacillus subtilis*, bacteriophage

## Abstract

Background: *Escherichia coli* and *Salmonella* contamination in goose bath water releases endotoxins like lipopolysaccharide (LPS), compromising immunity and hindering goose farming. Objective: This study evaluated effects of dietary *Bacillus subtilis* and bacteriophage supplementation on water quality, carcass traits, and muscle growth in Magang geese. Method: A total of 288 geese were divided into four groups based on similarity in weight (n = 6 geese): A (basal diet); B (basal diet + bacteriophage: 5.0 × 10^10^ PFU/L at 1:1000 dilution); C (basal diet + *Bacillus subtilis*: 5.0 × 10^9^ CFU/kg); D (basal diet + bacteriophage + *Bacillus subtilis*). Results: Supplementation significantly increased wing length, tibia length, and live weight at 60 days. It reduced water and plasma endotoxin levels and suppressed viable counts of *Escherichia coli*, *Salmonella*, and total bacteria in water across rearing stages. Supplementation up-regulated mRNA and protein expression of myogenic regulators (*MYOD*, *MYOG*, *MYH1*) and *IGF-1*, while down-regulating pro-inflammatory cytokines (*TNF-α*, *IL-6*), suggesting enhanced myofiber growth. Conclusion: These findings demonstrate that *Bacillus subtilis* and bacteriophage supplementation improves goose growth performance and immune status by modulating key genes, reducing pathogens and endotoxins, offering an eco-friendly strategy to enhance productivity and potentially reduce antibiotic dependency.

## Simple Summary

*Escherichia coli* and *Salmonella* contamination in goose bath water releases endotoxins like lipopolysaccharide (LPS), compromising immunity and hindering goose farming. Objective: This study evaluated dietary supplementation with *Bacillus subtilis* and bacteriophage on Magang geese. A total of 288 geese were divided into four groups based on similarity in weight (*n* = 6 geese): A (basal diet); B (basal diet + bacteriophage: 5.0 × 10^10^ PFU/L at 1:1000 dilution); C (basal diet + *Bacillus subtilis*: 5.0 × 10^9^ CFU/kg); D (basal diet + bacteriophage + *Bacillus subtilis*). Supplementation significantly increased wing length, tibia length, and live weight at 60 days. It reduced water and plasma endotoxin levels and suppressed viable counts of *Escherichia coli*, *Salmonella*, and total bacteria in water across rearing stages. Supplementation up-regulated mRNA and protein expression of myogenic regulators (*MYOD*, *MYOG*, *MYH1*) and *IGF-1*, while down-regulating pro-inflammatory cytokines (*TNF-α*, *IL-6*), suggesting enhanced myofiber growth. These findings demonstrate that *Bacillus subtilis* and bacteriophage supplementation improves goose growth performance and immune status by modulating key genes, reducing pathogens and endotoxins, offering an eco-friendly strategy to enhance productivity and potentially reduce antibiotic dependency.

## 1. Introduction

China produced 530 million meat geese in 2022, which is the country with the largest number of meat geese in the world according to data provided by China animal Husbandry association and the world Food and Agriculture Organization (FAO) [1]. Today, semi-intensive and intensive feeding systems are more profitable than traditional feeding methods [2,3]. In traditional feeding methods, after waterfowl excreta are discharged into the water, with the prolongation of breeding time and the accumulation of feces, Gram-negative bacteria in the water enzymatically degrade nitrogen and phosphorus from feces into ammonia and phosphate, which are rapidly absorbed, thus achieving a continuous and explosive multiplication in the contaminated water column. Bacterial death releases endotoxin (LPS), and geese living in such environments can be adversely affected by the accumulation of large amounts of endotoxin in their bodies during bathing and watering that cannot be completely removed. Semi-intensive and intensive feeding systems are characterized by a high degree of amenities, automation, and more scientific and effective disease prevention and control, reducing the probability of disease occurrence, and relatively high breeding density, more breeding numbers per unit area, and higher economic benefits [4]. Effective environmental control of waterfowl farming is particularly important as the demand for waterfowl products continues to grow with the increase in population. It is now widely accepted that the main cause of these problems is increased level of bacteria and lipopolysaccharide (LPS) in the goose rearing environment.

Skeletal muscle development in geese comprises a multistage process involving myogenic precursor cell proliferation, myoblast cell cycle exit, differentiation, and fusion into multinucleated myofibers. This developmental progression exhibits phase-specific characteristics across distinct growth periods. Myoblasts undergo terminal differentiation to form nascent myotubes, which subsequently mature into functionally specialized myofibers [5]. During primary myogenesis, myoblasts proliferate and fuse with each other to form primary myofibers [6]. This process is governed by the appropriate temporal expression of the myogenic regulatory factors (MRF). A second wave of myogenesis leads to the formation of secondary fibres, using primary muscle fibres as a scaffold. During the late embryonic period, muscle growth occurs through hypertrophy, and muscle fibre number is fixed at hatch with no post-hatch hyperplasia [7]. Skeletal muscle development in geese varies at different periods. The number of myofibers in waterfowl skeletal muscle is essentially determined at the embryonic stage; the acquisition of muscle mass after hatching is largely dependent on protein deposition and reflected in the thickening and growth of myofibers [8]. Myofiber density was the highest at 1 d and gradually decreased with time, while myofiber diameter increased. The diameter and cross-sectional area of myofibers increased gradually, and the density decreased gradually, but the number of myofibers contained in the muscle bundles did not change greatly [9].

Skeletal muscle growth and development is regulated by multiple genes. Several myogenic regulatory factors (MRFs) are associated with myogenesis, including *Myf5*, *MYOD*, *MYOG*, and *MYH1*. It is widely recognized that *Myf5* and *MYOD* are early factors in satellite cell myogenesis and determine whether muscle satellite cells can be activated to become myoblasts with myogenic properties. In addition to this, much evidence supports the notion that *MYOG* plays an important role in the differentiation of myoblasts and the maintenance of myofiber homeostasis while *MYH1* assumes the role of encoding the *MYH1* protein. Insulin-like growth factor-1 (*IGF-1*) is one of the best-characterized growth factors, and it has been shown to modulate muscle size and play a critical role in regulating muscle function [10]. Several lines of evidence indicate that cytokines such as interleukin-6 (*IL-6*) and tumour necrosis factor-α (*TNF-α*) are important regulators of muscle protein balance [11]. *TNF-α* impairs muscle protein metabolism when administered to control animals [12]. *IL-6* supports satellites cell proliferation and differentiation [13].

LPSs are bacterial surface glycolipids, produced by Gram-negative bacteria. It is present in the outer membrane of most Gram-negative bacteria, which stimulates the secretion of immune elements. Direct excretion of geese into water can increase bacterial and endotoxin levels, which in turn leads to the accumulation of endotoxins in geese, resulting in disease and reduced performance. Previous studies have shown that LPS injection inhibited growth performance of broilers and led to an inflammatory response, which negatively affected several parameters including splenic and bursal relative weight [14]; meanwhile, LPS significantly induced inflammatory cytokine production in both proliferation and differentiation stages of goose myoblasts [5].

*Bacillus subtilis* serves as a facultative anaerobe and is widely used as a possible candidate in monogastric feed due to high resistance of its spores to the harsh environment (such as the gastrointestinal tract of animals) and the possibility of long-term storage at ambient temperature [15]. It has been shown that *Bacillus subtilis* has a growth-promoting effect and can effectively improve the growth performance, immunity, and intestinal morphology of poultry [16,17,18,19].

Bacteriophages are probably the most abundant virus and are a group of biological entities with a genome consisting either of DNA or RNA and encapsulated in a protein coat (capsid) [9]. Dietary addition of bacteriophage increased the amount of *Lactobacillus* and *Bifidobacterium* and decreased the amount of *Salmonella* and *Coliform* in the fecal microbiota of growing pigs [16]. Previous studies about the application of *Bacillus subtilis* and bacteriophage mainly concentrated on the effects of dietary *Bacillus subtilis* or bacteriophage supplementation on intestinal morphology and gut microbiota [20,21,22,23]. Animal carcass traits is an index reflecting the growth and development of animals. However, there is still limited information on the influence of *Bacillus subtilis*, bacteriophage, and their combination on water quality, carcass traits, and muscle growth during waterfowl rearing. The specific mechanism by which *Bacillus subtilis* or bacteriophages improve waterfowl muscle function remains elusive.

We hypothesize that both *Bacillus subtilis* and bacteriophage may improve the growth performance and immune status of geese by reducing pathogens and endotoxins. *Bacillus subtilis* may enhance immune function to promote bacteriophage activity, or the reduction in pathogens by bacteriophage may enable *Bacillus subtilis* to colonize more effectively, thereby synergistically influencing the expression of myogenesis-related factor gene and improving the slaughter performance of geese. Thus, in the present study, we aimed to clarify the effect of *Bacillus subtilis* and bacteriophage individually or in combination on slaughtering performance, breeding water, and serum endotoxin, as well as effects on serum inflammatory cytokine and myogenesis-related factor gene expression and protein expression, which would in turn improve the productivity and efficiency of goose farming.

## 2. Materials and Methods

### 2.1. Animal Ethics

The experimental procedures strictly adhered to the ethical guidelines established by the Animal Care Committee of Zhongkai University of Agriculture and Engineering, with all protocols reviewed and approved by the institution’s Animal Experiment Committee (NO. 2021112709). Measures were rigorously implemented to minimize discomfort and distress in the study subjects. To ensure minimal distress, geese were euthanized via rapid exsanguination (cervical vessel transection) without prior anesthesia, as this method is recognized for immediate unconsciousness due to rapid cerebral hypoperfusion.

### 2.2. Animals and Management

One-day-old Magang goslings were procured from Guangdong Lvfengyuan Modern Agriculture Development Co., Ltd, Qingyuan, China Geese were raised under net-floor rearing conditions from day (d) 1 to 20, followed by a semi-dryland system from d 21 to 90, with a total trial duration of 90 days, and maintained with unrestricted access to water and standardized feed (GB/T 18823-2010) [24]. The feeding trial was conducted in September–October, and the ambient temperature was maintained at around 39 °C for yard feeding from 1 to 20 days old, and around 30–35 °C for semi-dry feeding from 21 to 90 days old. The composition of feed ingredients for Magang geese during the experimental period was maize, wheat, soybean meal, vegetable meal, sodium chloride, calcium hydrogen phosphate, L-lysine sulphate, DL methionine, and vitamins. In addition, we determined the main nutritional components of the diet, including crude protein (using the Kjeldahl nitrogen determination method, referring to GB/T 6432-2018) [25], crude fibre (using the filter bag method, referring to GB/T 6434-2022) [26], crude ash (using the muffle furnace calcination method, referring to GB/T 6438-2007) [27], calcium (using atomic absorption spectrometry, refer to GB/T 6436-2018) [28], total phosphorus (using molybdenum blue colorimetric method, refer to GB/T 6437-2018) [29], sodium chloride (using Folhard method, refer to GB/T 6439-2007) [30], and lysine (using pre-column derivatization -HPLC method, refer to AOAC 994.12) [31]. Specific nutritional levels are shown in Table 1. The bacteriophage used in the present study was a commercial product from Green-Agr Biotechnology Co., Ltd. Wuhan, China, consisting of a mixture of bacteriophage in a ratio of 1:1, targeting *Escherichia coli* and *Salmonella*. *Bacillus subtilis* was purchased from Huizhou Huinong Bio-technology Co., Ltd. The live bacteria number > 5 × 10^10^ CFU/g. All geese were vaccinated with Goose Plague Vaccine. In order to investigate the effects of *Bacillus subtilis* and bacteriophage on the growth period of geese, we collected samples from days 1 to 60 for analysis.

### 2.3. Experimental Design

In this experiment, a total of 288 healthy Magang geese (1 day) were selected and randomly divided into 4 experimental groups (72 geese in each group), and each group was further divided into 6 repeat pens, with 12 geese in each repeat pen. For the specific slaughter performance analysis and tissue sampling conducted on days 1, 30 and 60, a subset of 6 geese per group (n = 6/group/timepoint) was randomly selected from the total pool of 72 in each group. Samples were stored and transported in liquid nitrogen.

### 2.4. Water and Blood Sample Procedure

Blood specimens were obtained from six randomly selected geese per group on days 1, 30 and 60 for serum isolation and subsequent analytical procedures. Water samples were concurrently collected from the avian bathing pool on days 30 and 60. At each sampling event, 100 mL aliquots were drawn from five predefined locations (four peripheral corners and central region) within the pool at depths of 10–20 cm. These subsamples were homogenized to form a composite 500 mL representative sample per timepoint.

### 2.5. Slaughtering Performance

In total, 24 geese (6 geese per group, 3 male and 3 female) were randomly selected and fasted for 12 h with access to water before slaughtering on 1, 30, and 60 d. Then, 6 geese per group were weighed to record live weight and measure wing and tibial length. On 60 d, after slaughtering and releasing the blood, the geese were de-feathered and their slaughter weight was recorded. The whole body, including heart, liver, kidneys, glandular stomach, myogastrics exfoliated membranes and contents, surrounding fat, as well as lung, were measured and recorded as their half-eviscerated weight. Then, the visceral tissue was removed, and the carcass weight was recorded. The breast muscles and leg muscles were removed and their weights were recorded.

Dressed percentage = (slaughter weight/live weight) × 100%

Half-eviscerated weight percentage = (half-eviscerated weight percentage/live weight) × 100%

Carcass weight percentage = (carcass weight percentage/live weight) × 100%

Breast muscle weight percentage = (Breast muscle weight/carcass weight) × 100%

Leg muscle weight percentage = (Leg muscle weight/carcass weight) × 100%

### 2.6. Bacterial Colony Culture

Precisely 33 g Nutrient Agar (NA), 36 g Luria–Bertani (LB) agar, 50 g MacConkey agar, and 58.5 g SS agar powders were resuspend in 1 L deionized water. The mixture container was then securely capped, and the mixture was agitated thoroughly until complete dissolution, and the sterilized via autoclaving (121 °C, 15 min). After cooling to 50 °C, the liquefied media was aseptically dispensed into pre-sterilized Petri dishes and allowed to solidify under ambient conditions. For microbial quantification, water samples were serially diluted 10^−3^–10^−4^ times under laminar flow conditions. Aliquots (100 µL) of each dilution were spread uniformly across agar surfaces using sterile L-shaped glass rods. Plates were incubated upright for 10–20 min at 37 °C to absorb residual moisture, then inverted and cultured aerobically (37 °C, 18–24 h). Post-incubation, discrete colonies were quantified via standardized plate-count methodology, with colony-forming units (CFU) per plate systematically recorded.

### 2.7. Endotoxin Determination

Endotoxin levels in serum on days 1, 30, and 60 and water samples on day 30 and 60 were quantified using horseshoe crab lysate reagent (BIOENDO, Fujian, China). Serial dilutions of the endotoxin calibrant were prepared to generate a standard concentration series, which were then combined with the lysate reagent following manufacturer-specified ratios. Reaction mixtures were incubated under controlled thermal conditions (37 °C, 10–15 min), and absorbance values for each standard were measured at the prescribed wavelength using a Thermo Fisher enzymatic microplate reader. A calibration curve was constructed by plotting endotoxin concentrations (logarithmic scale) against their corresponding absorbance values (linear scale). Negative controls containing only reagent and buffer were processed in parallel to account for background interference. Test samples were diluted iteratively based on their matrix properties and projected endotoxin levels. Diluted samples were mixed with reagent as per protocol, incubated under identical thermal parameters as the standards, and promptly analyzed for absorbance at the target wavelength. Final endotoxin concentrations were derived by interpolating sample absorbance values against the standard curve, with results adjusted by subtracting the blank control’s baseline absorbance.

### 2.8. Sample Collection and H&E Staining

At days 1, 30 and 60, tissue blocks of the breast muscle superficialis muscle and leg muscle were removed, which were still attached to the bone, were cross-sectioned perpendicular to the orientation of the myofibers, and the cross-sectional area was traced on a transparent paper [5]. The sampling site for the leg muscle is the gastrocnemius muscle, and the sampling site for the breast muscle is a cross-section of the entire muscle. Skeletal muscle from 6 geese of 1, 30, and 60 d were utilized. Sections were processed by hematoxylin and eosin staining. A 20.0× image of muscle tissue was captured for each section on CaseViewer 2.2 scanning and viewing software. The micrographs were taken with the Axio Imager Z1 (ZEISS), zooming in 400× for each area. One field of view was selected for each of the three biological geese of each species in each period for the statistics. Then, 3 randomly selected fields of view per slice from 6 slices were photographed for each section, 6 myofibers or muscle bundles were randomly selected for each view, and the diameter of the myofibers (μm) or the cross-sectional area of the muscle bundles (μm^2^) was measured and averaged. The number and diameter (μm) of all myofibers in the field of view of the section were counted using Adobe Photoshop 2021, and the density was calculated by the number and field area of the section. The total number of myofibers was calculated by myofiber density and cross-sectional area.

### 2.9. RNA Extraction and Real-Time Quantitative PCR

Total RNA was isolated following the Trizol (Thermo Fisher, Waltham, MA, USA) protocol. RNA purity and concentration were quantified via full-wavelength spectrophotometry after resuspension in DEPC-treated water. cDNA synthesis utilized the ReverTra Ace qPCR RT Master Mix with gDNA Remover (TOYOBO, Osaka, Japan). Purity was assessed using spectrophotometry and the A260/A280 ratio was used to detect protein contamination. Values < 1.8 indicate the presence of protein/phenol contamination. RT-qPCR primers were designed using Primer Premier 5.0 (Table 2) through alignment with NCBI GenBank sequences. GAPDH served as the endogenous control, which is expressed in high abundance in muscle tissue and is easy to detect with low CT values, and its expression was found to be generally not significantly affected by treatments in muscle in our previous study. SYBR Select Master Mix (Thermo, USA) was prepared on ice for 20 µL reaction assemblies. Relative mRNA levels were determined via the 2^−∆∆CT^ method.

### 2.10. Western Blotting

Protein lysates underwent electrophoretic separation via SDS-PAGE and were subsequently electrophoretically transferred onto PVDF membranes. Membranes were blocked for 1 h with 5% skim milk in PBST (phosphate-buffered saline with 0.1% Tween-20). Primary antibodies included anti-MYOD (1:1000, Abcam ab16148, Cambridge, UK), anti-IL-6 (1:1000, Wanlei WL02841, Shenyang, China), anti-TNF*-α* (1:1000, Wanlei WL01581, China), anti-*MYH1* (1:10,000, Sigma M4276, St. Louis, MO, USA), and anti-GAPDH (1:10,000, Abcam ab181602, UK), which were applied in PBST at 4 °C overnight. Following PBS-T washes, membranes were probed with HRP-conjugated secondary antibodies—goat anti-mouse IgG (1:10,000, Abcam ab97023, UK) or goat anti-rabbit IgG (1:10,000, Wanlei WLA32023a, China)—for 1 h at ambient temperature. A secondary incubation step was performed at 37 °C for 1 h to enhance antigen–antibody binding. Chemiluminescent signals were generated using an ECL substrate (Beyotime, Beijing, China) and imaged on a Tanon-200 Multi documentation system (Tanon, Shanghai, China). Quantitative densitometry was executed via ImageJ software (v1.8.0) to assess band intensity.

### 2.11. Statistical Analysis

Six biological geese were set in each group, normality was assessed using the Shapiro–Wilk test, with *p* ≥ 0.05 indicating that the data met the assumption of normality. For homogeneity of variances, Levene’s test was employed, where *p* ≥ 0.05 supported homogeneity of variances across groups, and Tukey’s Honestly Significant Difference (HSD) was used for post hoc comparisons when ANOVA detected significance. *p* > 0.05 means that the difference is not significant, *p* < 0.05 means that the difference is significant.

## 3. Result

### 3.1. Effect of Bacillus subtilis and Bacteriophage on Slaughtering Performance

To determine whether dietary supplementation with *Bacillus subtilis* and bacteriophage affected goose slaughtering performance, tibial length and wing length were measured. Body weight, half-eviscerated weight, eviscerated weight, and their ratio to body weight were analyzed, as well as breast muscle weight, leg muscle weight, breast muscle weight rate, and leg muscle weight rate. From previous work, ADFI results showed that there were no significant differences (*p* > 0.05) among groups [22].

**Figure 1:**
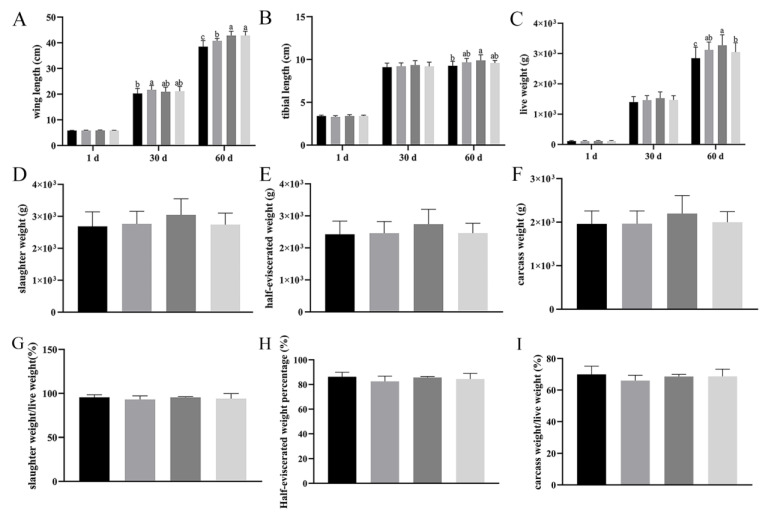
Effect of *Bacillus subtilis* and bacteriophage on slaughtering performance. (**A**) Wing length at 1, 30, and 60 d. (**B**) Tibial length at 1, 30, and 60 d. (**C**) Live weight at 1, 30, and 60 d. (**D**) 60 d slaughter weight. (**E**) Statistical results of 60 d half-eviscerated weight. (**F**) 60 d carcass weight. (**G**) Statistical results of slaughter weight percentage at 60 d. (**H**) Statistical results of half-eviscerated weight percentage at 60 d. (**I**) Statistical results of carcass weight percentage at 60 d. Black—control group; grey—bacteriophage group; dark grey—*Bacillus subtilis* group; light grey—mix group. Values with different superscript letters are significantly different (*p* < 0.05).

At 30 d, there was a significant difference in wing length between Group B and A (*p* < 0.05). At 60 d, Group B, C, and D were significantly higher than group A, while group C and D were also significantly higher than group B (*p* < 0.05) (Figure 1A).

The tibial length of group C was significantly higher than those of group A, B, and D on 60 d, while groups B and D showed no difference from group A (*p* < 0.05) (Figure 1B).

Live weight increased with age. Although no significant differences existed at 1 and 30 d (*p* > 0.05), the live weights of group B, C, and D were significantly higher than those of group A at 60 d, with group C being the highest and group B the second highest (*p* < 0.05) (Figure 1C).

At 60 d, no significant differences occurred among groups in slaughter weight, half-eviscerated weight, carcass weight, or their corresponding percentage (*p* > 0.05). Although significant differences were not reached, the slaughter weight, half-eviscerated weight, and carcass weight of bacteriophage-added group showed non-significant increase (Figure 1D–I).

**Figure 2:**
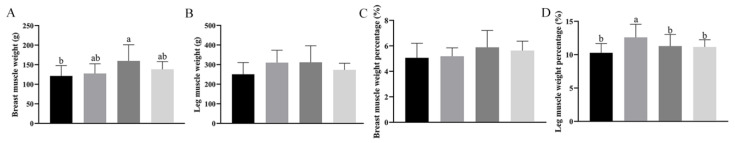
Effect of *Bacillus subtilis* and bacteriophage to skeletal muscle weight and its ratio to slaughter weight. (**A**) Breast muscle weight at 60 d. (**B**) Leg muscle weight at 60 d. (**C**) Statistical results of breast muscle weight percentage at 60 d. (**D**) Statistical results of leg muscle weight percentage at 60 d. Black—control group; grey—bacteriophage group; dark grey—*Bacillus subtilis* group; light grey—mix group. Values with different superscript letters are significantly different (*p* < 0.05).

We further explored the effect of adding *Bacillus subtilis* and bacteriophage individually or in combination on skeletal muscle we measured breast muscle weight, leg weight, breast muscle weight percentage, and leg weight percentage on day 60. It can be seen that breast muscle weight was significantly higher in group C than in groups A, B, and D (*p* < 0.05) (Figure 2A). Although the breast muscle weight percentage in group C was numerically higher than that in group A, it did not show a significant difference (*p* > 0.05) (Figure 2C). The leg weight percentage was significantly higher in group B than in groups A, C, and D (*p* < 0.05) (Figure 2D), and no significant differences were shown between the leg muscle weights of the different treatment groups, although the leg weight in group B and C was numerically higher than in group A and D (*p* > 0.05) (Figure 2B).

These results suggested that dietary *Bacillus subtilis* and bacteriophage supplementation, individually or combined, improved some of the slaughter performance of the geese, especially on day 60, and dietary supplementation with *Bacillus subtilis* or bacteriophage alone may have a greater effect on the muscle than a mixture of the two. These findings indicated that dietary supplementation with *Bacillus subtilis* and bacteriophage, especially bacteriophage, can significantly increase the body weight, breast muscle weight, and leg muscle weight, as well as the percentage increase in tibial length and wing length, to improve production performance of 60-day-old Magang geese. Even though there were no significant differences in overall body weight or total production, the significant increase in percentage of muscle suggests that the supplementation improved the efficiency of nutrient partitioning, allowing more nutrients to be used for the deposition of muscle mass rather than fat or other tissues.

### 3.2. Effect of Dietary Supplementation with Bacillus subtilis and Bacteriophage on Bacteria in Bath Water

In order to explore the bacterial effects of *Bacillus subtilis* and bacteriophage on the bath water of Magang geese, we examined the levels of bacterial total colony, *Escherichia coli*, and *Salmonella* in the bath water at 30 and 60 d.

**Figure 3:**
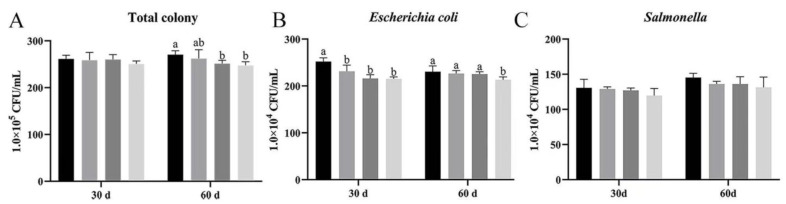
Effect of dietary supplementation with *Bacillus subtilis* and bacteriophage on bacteria in bath water. (**A**) Total colony content at 30 and 60 d. (**B**) *Escherichia coli* content at 30 and 60 d. (**C**) *Salmonella* content at 30 and 60 d. Black—control group; grey—bacteriophage group; dark grey—*Bacillus subtilis* group; light grey—mix group. Values with different superscript letters are significantly different (*p* < 0.05).

As it shown, the total bacterial colony on day 30 was not significantly different among the groups, with group D being slightly lower than group A (*p* > 0.05). At day 60, groups B, C, and D had lower total bacterial colony as compared to group A, while those of groups B and C were significantly lower than those of group A (*p* < 0.05) (Figure 3A).

Group D had lower total bacterial colony content on day 30, but showed no significant differences. At 60 d, group A had the highest total bacterial colony counts among all groups, while those on groups C and D were significant lower (*p* < 0.05) (Figure 3A).

*Escherichia coli* content was significantly lower in treatment groups (B, C, D) than in group A at 30 d, and significantly lower in group D than in groups A, B, and C at 60 d (*p* < 0.05) (Figure 3B).

Although there was no significant difference in *Salmonella* concentration at both 30 and 60 d, *Salmonella* concentration in groups B, C, and D were slightly lower than in group A (Figure 3C).

These results showed that bacteriophage and *Bacillus subtilis* can significantly reduce the concentrations of specifically quantified *Escherichia coli* and *Salmonella* in water and improve the environment of bath water. Due to limitations in the specificity of the assay, co-reduction of other pathogens cannot be excluded, but the main effect should be attributed to bacteriophage and *Bacillus subtilis*.

### 3.3. Effect of Dietary Supplementation with Bacillus subtilis and Bacteriophage on Serum and Bath Water Endotoxin

In order to detect the effects of bacteriophage and *Bacillus subtilis* on farmed water and serum endotoxin in Magang geese, this paper examined bath water and serum endotoxin using horseshoe crab reagent colorimetric methods.

**Figure 4:**
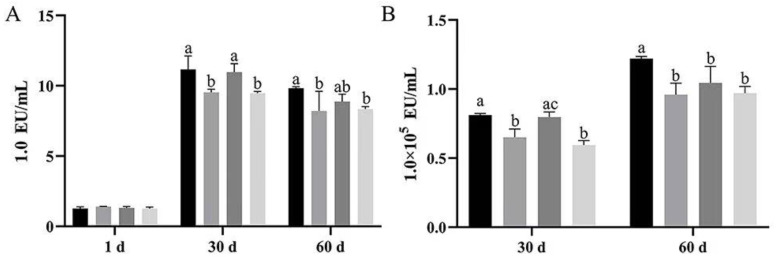
Results of endotoxin treatment of cultured serum and water quality by *Bacillus subtilis* and bacteriophage. (**A**) Results of serum endotoxin content. (**B**) Results of endotoxin content of bathing water. Black—control group; grey—bacteriophage group; dark grey—*Bacillus subtilis* group; light grey—mix group. Values with different superscript letters are significantly different (*p* < 0.05).

At 30 d, serum endotoxins were significantly lower in both Groups B and D than in group A and C (*p* < 0.05). Although there was no significant difference in group C compared to group A, the amount was less than group A (*p* > 0.05). At 60 d, serum endotoxins were significantly lower in both Group B and D than in group A and C (*p* < 0.05), and it was lower in group C than in group A without significant difference (*p* > 0.05) (Figure 4A).

Bath water endotoxins were significantly lower in both Group B and D than in group A and C. At 60 days, the amount in the treatment groups (B, C, D) were significantly lower than in group A (*p* < 0.05) (Figure 4B).

The results showed that bath water and serum endotoxin both increased with period and stabilized after 30 d. Dietary supplementation with *Bacillus subtilis* or the combination of *Bacillus subtilis* and bacteriophage was able to significantly reduce bath water and serum endotoxin at 30 and 60 d, but dietary supplementation with bacteriophage alone had less effect.

### 3.4. Effect of Dietary Supplementation with Bacillus subtilis and Bacteriophage on the Diameter and Density of Breast and Leg Myofiber

**Figure 5:**
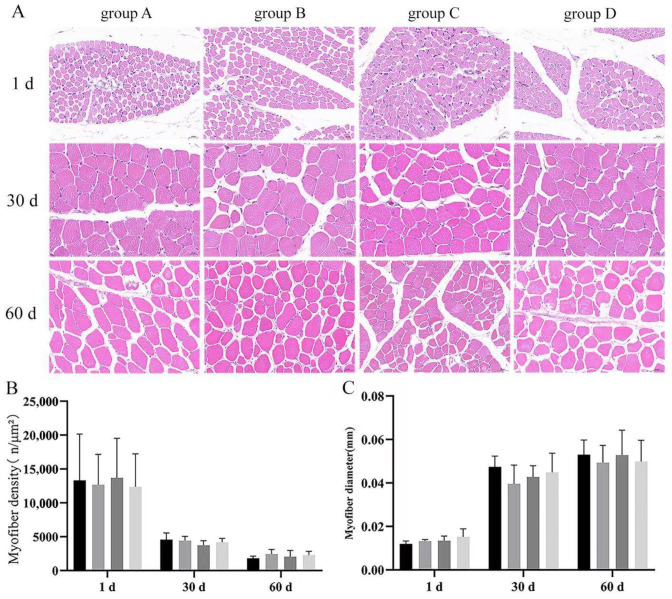
Results of H&E staining of the breast muscles of Magang goose at different periods. (**A**) Results of H&E staining of the leg muscles of Magang goose at different periods. (**B**) Statistical results of myofiber density. (**C**) Statistical results of myofiber diameter. Black—control group; grey—bacteriophage group; dark grey—*Bacillus subtilis* group; light grey—mix group. Values with different superscript letters are significantly different (*p* < 0.05).

**Figure 6:**
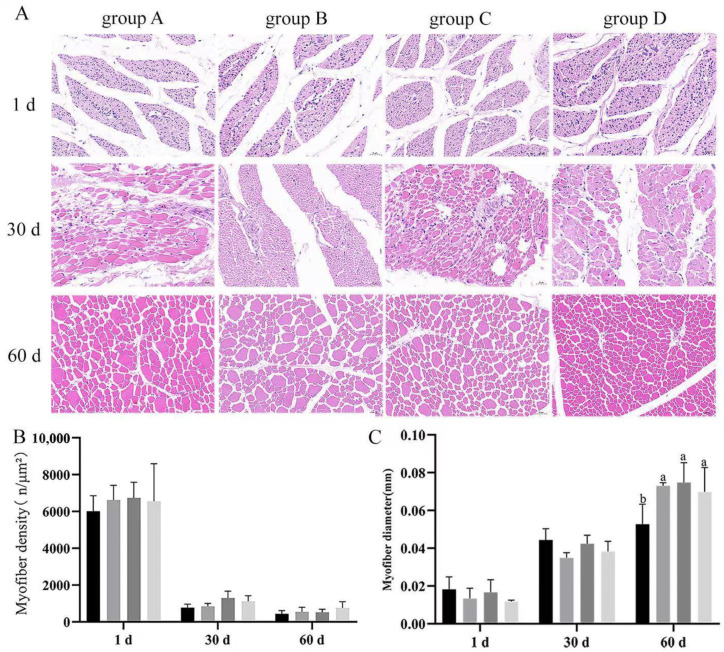
Results of H&E staining of the leg muscles of Magang goose at different periods. (**A**) Results of H&E staining of the leg muscles of Magang goose at different periods. (**B**) Statistical results of myofiber density. (**C**) Statistical results of myofiber diameter. Black—control group; grey—bacteriophage group; dark grey—*Bacillus subtilis* group; light grey—mix group. Values with different superscript letters are significantly different (*p* < 0.05).

Skeletal muscle is an important criterion for judging the performance of poultry production; to explore the difference between two treatments, we performed H&E-stained section analysis. Results indicated that there were no significant differences (*p* > 0.05) in the breast muscle density or diameter among groups at every timepoint (Figure 5A). There were no significant differences (*p* > 0.05) in the leg muscle density of the groups at every timepoint. Moreover, there were no significant differences in the leg muscle myofiber diameter at 1 and 30 d (*p* > 0.05) (Figure 5B); leg muscle myofiber diameters were significantly higher in the treated groups (B, C, D) than in the control group (group A) at day 60, with group C having the highest. These results suggested that *Bacillus subtilis* and bacteriophage may not be effective on the leg muscle until after the middle of the fattening period (*p* < 0.05) (Figure 6C).

### 3.5. Effect of Dietary Supplementation with Bacillus subtilis and Bacteriophage on Inflammation and Myogenic Genes

The mRNA expression of pro-inflammatory factors and myogenic genes was measured by real-time RT-qPCR at day 1, 30, and 60 after dietary supplementation with *Bacillus subtilis* and bacteriophage.

**Figure 7:**
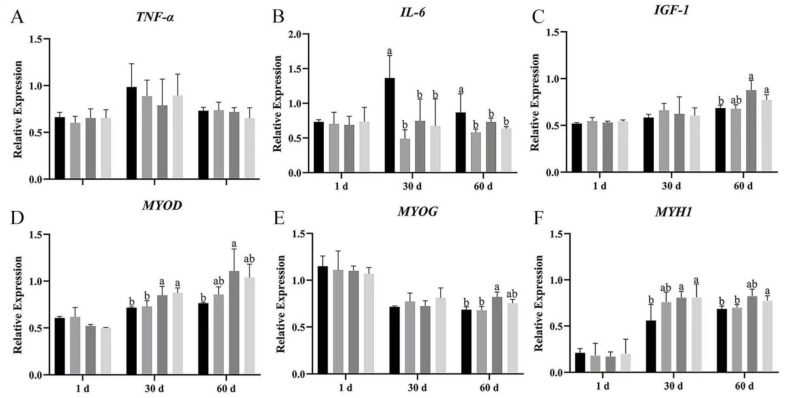
The mRNA relative expression of vital immunity and myogenic genes in breast muscle. The gene expression of (**A**) *TNF-α*, (**B**) *IL-6*, (**C**) *IGF-1*, (**F**) *MYH1*, (**D**) *MYOD*, and (**E**) *MYOG* in breast muscle. Black—control group; grey—bacteriophage group; thick grey—*Bacillus subtilis* group; light grey—mix group. Values with different superscript letters are significantly different (*p* < 0.05).

**Figure 8:**
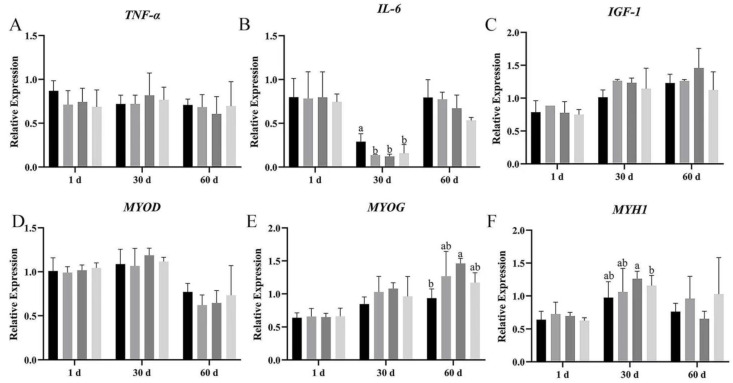
The mRNA relative expression of vital immunity and myogenic genes in leg muscle. The gene expression of (**A**) *TNF-α*, (**B**) *IL-6*, (**C**) *IGF-1*, (**F**) *MYH1*, (**D**) *MYOD*, and (**E**) *MYOG* in leg muscle. Black—control group; grey—bacteriophage group; thick grey—*Bacillus subtilis* group; light grey—mix group. Values with different superscript letters are significantly different (*p* < 0.05).

*TNF-α* mRNA expression showed no significant difference among the four groups on day 1, 30, and 60 of breast and leg muscle (Figure 7A and Figure 8A); however, *TNF-α* expression in groups B, C, and D was lower than in group A on breast muscle, meanwhile the mRNA expression of *TNF-α* was also more decreased in group C than in group A (*p* > 0.05). Dietary supplementation with *Bacillus subtilis* and bacteriophage individually or in combination significantly reduced *IL-6* mRNA expression on days 30 and 60 in breast muscles (Figure 8B). In leg muscles, *IL-6* mRNA expression was significantly higher in the control group than in the treatment groups on day 30 (*p* < 0.05) (Figure 8B), indicating that bacteriophage addition or a combination of both increased *IL-6* mRNA expression.

The expression of *IGF-1* mRNA difference in breast muscle was significantly higher in groups C and D than in groups A and B, at 60 d (Figure 7C); meanwhile, in leg muscle, bacteriophage-treated geese (group C) had higher *IGF-1* mRNA expression, but the difference was not statistically significant (*p* > 0.05) (Figure 8C).

We then explored the effects of *Bacillus subtilis* and bacteriophage individually or in combination on myogenic mRNA expression (*p* < 0.05). In addition, higher expression of *MYOG* was detected in both breast and leg muscle on day 60 (Figure 7E and Figure 8E). There was no difference in the mRNA expression level of *MYOD* in leg muscle (*p* > 0.05), but a significant difference occurred in breast muscle at 30 and 60 d, with group C having the highest mRNA expression and group D having the second highest (*p* < 0.05) (Figure 7D and Figure 8D). *MYH1* mRNA expression differed significantly between combination addition (group D) and control (group A) at both 30 and 60 d (*p* < 0.05). Bacteriophage addition (group C) showed a significant difference at 30 d (*p* < 0.05) and higher mRNA expression at 60 d without statistical significance (*p* > 0.05) (Figure 7F and Figure 8F). These results suggested that the molecular mechanism by which *Bacillus subtilis* promotes muscle production might not be consistent with bacteriophage.

Furthermore, although *MYOD* expression did not differ across periods among the four groups (*p* > 0.05), *MYH1* expression in group D was significantly higher than in group A, while *MYOG* was significantly higher than in groups A, C, and D (*p* < 0.05). These results suggested that dietary supplementation with *Bacillus subtilis* and bacteriophage could possibly enhance the expression of myogenic factors.

The results demonstrated that dietary supplementation with *Bacillus subtilis* and bacteriophage can decrease the expression of inflammatory factors and increase the expression of myogenic factors. Longer treatment with *Bacillus subtilis* and bacteriophage individually or in combination might lead to more efficient results.

### 3.6. Effect of Bacillus subtilis and Bacteriophage on Inflammation and Myogenic Proteins

To illustrate the effects of dietary supplementation with *Bacillus subtilis* and bacteriophage on the expression level of inflammatory cytokines and myogenic proteins in Magang geese, Western blot analysis was used to evaluate IL-6 (inflammatory cytokine), *MYOD*, and *MYH1* expression in both breast and leg muscles at different rearing stages (0, 30 and 60 days).

**Figure 9:**
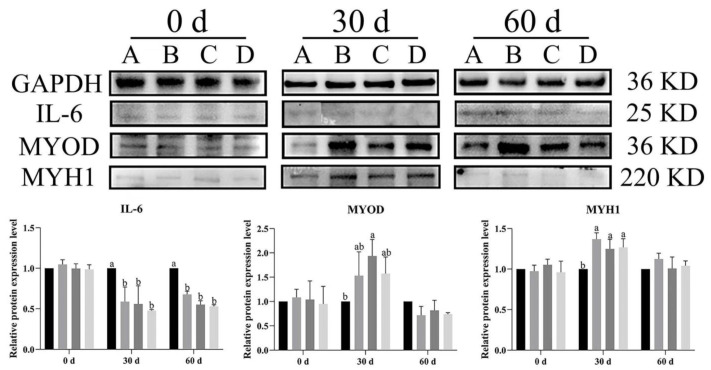
The Western blot picture and quantitative results of vital immunity and myogenic genes in breast muscle. Black—control group; grey—bacteriophage group; dark grey—*Bacillus subtilis* group; light grey—mix group. Values with different superscript letters are significantly different (*p* < 0.05). The original Western blot picture can be found in Figure S1.

**Figure 10:**
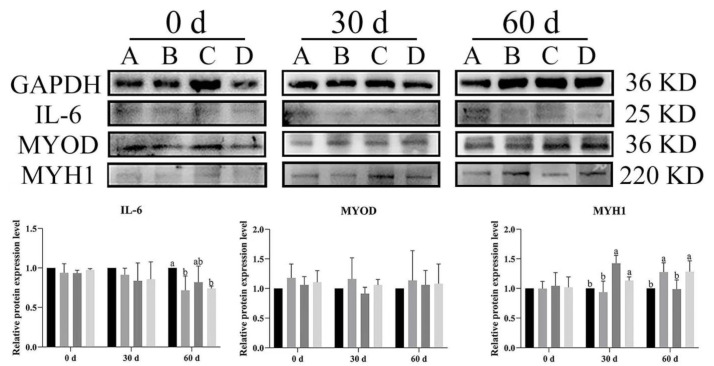
The Western blot picture and quantitative results of vital immunity and myogenic genes in leg muscle. Black—control group; grey—bacteriophage group; dark grey—*Bacillus subtilis* group; light grey—group. Values with different superscript letters are significantly different (*p* < 0.05). The original Western blot picture can be found in Figure S2.

In breast muscle, *IL-6* expression levels were significantly lower across time in the treatment groups (B, C, D) than in control group (A) at both 30 and 60 d (*p* < 0.05) (Figure 9). Similarly, at day 60, *IL-6* was significantly higher expression in group A of leg muscles as compared to other groups (*p* < 0.05) (Figure 10). *MYOD* expression was higher in the treatment groups than in the control group, with group C (*Bacillus subtilis*) showing significantly higher expression at 30 d (*p* < 0.05). Additionally, significant higher expression was noted in groups B, C, and D as compared to group A (*p* < 0.05). Likewise, *MYH1* expression had a similar trend to *MYOD*, especially at 30 d, and *MYH1* expression was significantly higher in groups B, C, and D compared to in group A (*p* < 0.05) (Figure 10). In the leg muscles, *MYH1* expression was significantly higher in groups C and D than in groups A and B at 30 d (*p* < 0.05), and in groups B and D than in A and C at 60 d (*p* < 0.05) (Figure 10).

The results showed that dietary supplementation with *Bacillus subtilis* and bacteriophage or their combination decreased the expression of *IL-6* and increased the expression of *MYOD* and *MYH1* in both breast and leg muscle. Dietary supplementation with *Bacillus subtilis* and bacteriophage or their combination had a similar effect of reducing *IL-6* protein expression and dietary supplementation with bacteriophage was more effective than dietary supplementation with *Bacillus subtilis* in increasing the expression of myogenic factors *MYOD* and *MYH1* protein.

## 4. Discussion

In this study, we found that dietary supplementation with *Bacillus subtilis* and bacteriophage improved the growth performance of Magang geese, reduced the total number of bacterial total colony of *Escherichia coli* and *Salmonella*, improved the environment of the bath water, and enhanced the immune system, contributing to the development of the breast and leg muscles by decreasing the inflammatory response as well as promoting the expression of myogenic factors.

### 4.1. Dietary Supplementation with Bacillus subtilis and Bacteriophage Improves Geese Growth Performance

With the growing interest in probiotics and their application in animal research, mounting evidence supports the concept that dietary supplementation with *Bacillus subtilis* or bacteriophage could promote the growth performance of poultry [16,22,23,26,27,28,29,30,31,32,33,34]. Several reports have shown that probiotics have no significant effect on body weight gain; dietary supplementation with *Bacillus subtilis* increased the weight gain of broiler chicks only in the early stages of life, while there was no difference in weight gain during the fattening period [33,34]. Moreover, other studies have shown that dietary *Bacillus subtilis* supplementation can improve the growth parameters even under conditions of heat stress [35] and body weight gain was also improved by the addition of bacteriophages into the diet [21,22]. Consistent with these studies, our results demonstrated that, during the breeding period, supplementation with *Bacillus subtilis* and bacteriophage in geese diets possesses the potential to improve the slaughter performance of geese by increasing body weight, wing length, tibia length, breast muscle weight, and leg muscle percentage. Dietary supplementation with *Bacillus subtilis* in our study also does not seem to improve body weight gain, in disagreement with those reported bacteriophage can improve body weight.

### 4.2. Dietary Supplementation with Bacillus subtilis and Bacteriophage Prevents Colonization and Reduces Proliferation of Harmful Bacteria

It is widely accepted that the main reason for dietary supplementation with *Bacillus subtilis* and bacteriophage to improve growth performance is colony regulation.

*Bacillus subtilis* is a spore-forming probiotic bacterium that competes with pathogenic bacteria by vying for nutrients and attachment sites in the gut, thereby inhibiting the growth of harmful bacteria. It creates a more favourable growth environment for other beneficial bacteria and can regulate the host’s immune system, reducing low-grade inflammation caused by pathogenic bacteria and improving nutrient utilization. Bacteriophage directly lyse specific pathogenic bacterial strains, reducing their negative effects, and indirectly promote the growth of non-target, potentially beneficial bacteria by reducing the presence of pathogens. We speculate that *Bacillus subtilis* and bacteriophage have a mutually beneficial relationship.

In recent years, several reports have shown that *Bacillus subtilis* significantly reduced the *Salmonella* load in the intestinal tract of poultry as well as in the surrounding environment [36,37]; it can also competitively exclude *Escherichia coli* and *Campylobacter jejuni* in poultry. Moreover, research on bacteriophage therapy for bacterial diseases in livestock has been actively carried out. Bacteriophage therapy for *Salmonella*, *Escherichia coli*, *Campylobacter*, *Streptococcus*, and other pathogenic bacteria has been reported, which are not only pathogenic to livestock, but also harmful to humans [21,38,39,40,41,42,43]. In our study, dietary supplementation with *Bacillus subtilis* or bacteriophage also seemed to reduce *Escherichia coli* and total bacterial colony, and although there was no significant difference in *Salmonella* load, we have seen a downward trend in *Salmonella*. Although we did not quantitatively measure initial *Salmonella* colonization levels, all geese were from the same commercial farm with standardized management procedures. Although the reduction in *Salmonella* load by dietary supplementation was not significant, the consistent downward trend was consistent with the associated Gram-negative *Escherichia coli* trend, and we believe that extending the addition treatment time will lead to a significant difference. In the present study, dietary supplementation with *Bacillus subtilis* or bacteriophage also appeared to reduce *Escherichia coli* and total colony counts, but there was no significant difference in *Salmonella* counts. Consistent with our findings, previous studies have reported that dietary supplementation with bacteriophage improved the performance of broilers and laying hens while reducing the concentrations of *Escherichia coli* and *Salmonella* in feces [42]. We can assume that the lack of difference in *Salmonella* counts in this study may be due to the fact that dietary supplementation with *Bacillus subtilis* or bacteriophage works well to stop the infection of harmful bacteria and regulate the microbiota.

### 4.3. Dietary Supplementation with Bacillus subtilis and Bacteriophage to Diets Improves Growth Performance Possibly Due to Their Ability to Elicit an Immune Response and Effectively Reduce Inflammation

The immune system is closely related to growth performance. In the body, LPS commonly known as endotoxin, might cause natural or innate immune responses, leading to the elevation of inflammatory cytokines and a decrease in the immune function [43,44,45]. The activities of geese generally take place in water, so the pollution of farm water can have an impact at all periods of growth and development, and exogenous LPS pollution can lead to increased accumulation of endotoxins in the body, which can reduce its growth performance. In this study, dietary supplementation with *Bacillus subtilis* or bacteriophage or a combination of the two showed varying degrees of ability to reduce water endotoxin as well as serum endotoxin, especially *Bacillus subtilis* treatment and the combination of the two. This is consistent with the findings of previous studies that *Bacillus subtilis* or bacteriophage could alleviate the adverse effects on the growth performance of broilers challenged with LPS [46,47].

The cytokines in serum could reflect the inflammatory reaction of the body. Tumour necrosis factor alpha (*TNF-α*) is a crucial agent that modifies the immune response and the inflammatory-cell-induced-tissue-damage, while *IL-6* is generally deemed as a pro-inflammatory cytokine produced by classically activated macrophages [47].

Inflammation was negatively correlated with body weight in human and animal studies. The mRNA expression of inflammatory factors *TNF-α* and *IL-6* in the breast muscle were both reduced under *Bacillus subtilis* or bacteriophage dietary treatments, as in previous studies [45,46]. It has been shown that bacteriophage inhibit the expression of inflammatory factors in mouse [44,45] and that probiotic bacteria act as inhibitors rather than stimulators of pro-inflammatory responses against pathogenic bacteria [48]. It has also been reported that bacteriophage play a positive role in the reduction in pro-inflammatory factors in pigs [49] and the results of the present study showed that the mRNA expression of inflammatory factors in the leg muscles was reduced by treatment with bacteriophage diets or a combination of the two, which is in agreement with the previous study. On the contrary, it has been reported that the relative expression of inflammatory cytokines such as *IL-1b*, *IL-6*, *IL-10*, *TNF-a*, and *IFN* in the small intestinal mucosa was significantly up-regulated under the stimulation of recombinant bacteriophage [19]. The results of the present study showed that the mRNA expression of inflammatory factors in the breast muscles was reduced by dietary *Bacillus subtilis* supplementation, bacteriophage supplementation, or a combination of the two, which is in agreement with the previous study. Consistent with previous studies, *IL-6* expression in leg muscles was significantly increased by dietary addition of bacteriophage or *Bacillus subtilis* and bacteriophage combination. Even though IL-6 is a pro-inflammatory factor, increased levels of its mRNA expression may induce anti-inflammatory mediators to participate in negative feedback regulation, promoting repair and reducing inflammatory responses. In muscle development, genetic overexpression or addition of exogenous *IL-6* increases the expression of muscle-specific genes and supports their pro-muscle functions [50,51]. The expression of *IL-6* is synergistically related to myogenic factors such as *MYF5* and *MYOD* [52].Thus, its increased expression may have led to a reduced inflammatory response and promoted muscle development. In the present study, protein expression in leg muscles showed a similar trend, which suggests that *Bacillus subtilis* or bacteriophage have anti-inflammatory effects; however, the mechanisms of immune expression in breast and leg muscles are inconsistent and require further study.

### 4.4. Dietary Supplementation with Bacillus subtilis and Bacteriophage May Increases Breast and Leg Muscle Weight by Increased Expression of Immune Factors and Muscle-Forming Genes

Skeletal muscle development is regulated by myogenic regulators including *MYOD*, *MYOG*, and *MYH1* [47,53,54,55,56]. Dietary supplementation with *Bacillus subtilis* or bacteriophage or their mixture increased the expression levels of muscle-forming factors to varying degrees. *Bacillus subtilis* may have a stronger ability to regulate leg muscles than breast muscles, and dietary supplementation with bacteriophage or *Bacillus subtilis* alone may be more effective than their combination.

Dietary supplementation with bacteriophage or a mixture was able to increase the mRNA expression of the growth factor *IGF-1* in breast muscle. Previous studies concluded that there is a mutual synergism between the expression of *IGF-1* and *MYOD*, and that their up-regulation leads to the up-regulation of muscle-forming factors, which, in turn, promotes muscle growth, suggesting that the immune system and muscle growth performance are tightly linked [47,57].

Another possibility is that administration of *Bacillus subtilis* or bacteriophage could enhance the geese’ immunity, which in turn regulates skeletal muscle development [48,58]. Skeletal muscle development consists of two phases: myoblast proliferation and differentiation. A study had found that LPS reduced the differentiation ability of goose embryonic myoblasts [19]. Moreover, LPS promotes myotube apoptosis and inhibits the proliferation of myofibroblasts, which reduces muscle mass, inhibits myofiber regeneration, and ultimately leads to muscle atrophy [5]. In our study, the decrease in serum endotoxin and the increase in leg muscle myofiber diameter at day 60 after dietary supplementation with *Bacillus subtilis* or bacteriophage or a combination of both suggests that *Bacillus subtilis* and bacteriophage can play a role in regulating the proliferation and differentiation of adult myoblasts. Also, mRNA expression of myogenic factors and protein expression increased significantly, reinforcing this conclusion. This suggests that dietary supplementation with *Bacillus subtilis* or bacteriophage or a combination of both may enhance the proliferation and differentiation of myogenic cells and by enhancing immunocompetence.

Skeletal muscle development depends greatly on the development of myofibers. Myofibers are the basic constituent unit of animal muscle [59] that can be classified into four types according to the polymorphism of myosin heavy chain (*MYH1*): slow-oxidizing type (I), fast-oxidizing type (IIa), fast glycolytic type (IIb), and intermediate type (IIx), which are, respectively, encoded by myosin heavy chain (MyHC) I, IIa, IIx, and IIb isoform genes [60]. Regarding the effect of *Bacillus subtilis* supplementation on muscle production, the results of different studies have been inconsistent. Previous studies have shown that the addition of *Bacillus subtilis* to the diet may decrease the *MYH1* mRNA and protein expression [61]. It was clear from our study that the administration of *Bacillus subtilis* had a beneficial effect on myogenic factors, with a increased expression on both mRNA and protein in *MYOD* and *MYH1*, which is consistent with a previous study [62]. Current studies have basically investigated the effects of dietary supplementation with bacteriophage on some immune responses and intestinal gene expression, as well as the effects on bacteria [21,32,40,59,60,61,62,63,64,65,66], indicating that bacteriophage can improve the immune ability of the organism, reduce the inflammatory response, and play a role in decreasing bacterial colonization, but very few studies have been conducted to explore the role of bacteriophage on myogenic factors, and the present study found that bacteriophage played a role in promoting myogenic factors, even exceeding the effect of *Bacillus subtilis*, and the specific mechanism of this remains to be further explored. We hypothesize that *Bacillus subtilis* and bacteriophage may inhibit the proliferation of pathogenic bacteria such as *Escherichia coli* and *Salmonella* in the intestinal tract by competitive rejection and secretion of antimicrobial peptides and reduce the entry of lipopolysaccharide into the bloodstream, which reduces the activation of the TLR4/MyD88 signalling pathway in muscle tissues and alleviates systemic inflammation [67]. Additionally, we hypothesized that *Bacillus subtilis* and bacteriophage may also be activating certain metabolites through digestion, which in turn activate the AMPK/mTOR pathway in muscle tissues, inhibiting NF-κB-mediated inflammatory responses and reducing *TNF-α* and *IL-6* levels, while promoting protein synthesis and myofiber development [19].

While this study provides evidence for the beneficial effects of *Bacillus subtilis* and Bacteriophage on geese, several limitations should be acknowledged. Firstly, the absence of metabolite profiling such as assessing short-chain fatty acid levels in the gut limits our ability to definitively elucidate the mechanistic pathways underlying the observed effects. Secondly, our histological analysis was limited to breast and leg muscles, and a more comprehensive topographic examination could provide deeper insights. Future research should aim to incorporate metabolomic and more extensive histological analyses to directly measure the effects.

## 5. Conclusions

1. Dietary supplementation with *Bacillus subtilis* or bacteriophage or their combination promoted the growth performance of geese, significantly increased 60-day-old live weight, pectoral and leg muscle weight, and increased tibia and wing length.
2. Dietary supplementation with *Bacillus subtilis* or bacteriophage or their combination significantly reduced the levels of water and blood endotoxin in the feeding environment; inhibited the level of *Escherichia coli*, *Salmonella,* and total bacterial colony in water; and improved the quality of bath water.
3. Dietary supplementation with *Bacillus subtilis* or bacteriophage or their combinations reduced the expression of immune factors and increased the expression of myogenic genes, thereby thickening the diameter of myofibers and promoting myogenesis.

These findings could provide theoretical support for the use of *Bacillus subtilis* or bacteriophage instead of antibiotics and offer potential nutritional strategies to improve production performance and immune function in geese.

## Figures and Tables

**Table 1:** Nutritional level of Magang goose diet and basic dietary composition.

Nutrient	Content	Ingredient	Content (%)
Crude protein	12.0 ± 0.59	Corn	50
Crude fibre	12.0 ± 0.08	Wheat middlings	8
Crude ash	12.0 ± 0.09	Wheat bran	14
Calcium	0.89 ± 0.31	Soybean meal	10
Total phosphorus	0.75 ± 0.28	Rapeseed meal	5
Sodium chloride	0.63 ± 0.27	DDGS	4.55
Moisture	13.5 ± 0.13	Limestone	6
Lysine	0.5 ± 0.16	Dicalcium phosphate	0.9
		Sodium bicarbonate	0.7
		Salt	0.25
		Vitamin-mineral premix	0.6
		Total	100

**Table 2:** Primer sequences for RT-qPCR.

Primer Name	Forwad (5′-3′)	Reverse (3′-5′)	Annealing Temperature (°C)
*MYH1*	CTCCTCACGCTTTGGTAAAT	GCTCTGGCTTCTTGTTGGAC	55
*MYOD*	AAGGCGTGCAAGAGGAAGAC	TGGTTGGGGTTGGTGGA	55
*MYOG*	CCCGAGCACTGCCCCGGGCAAT	CGCTCCTGCTGGTTGAGGCTGCTG	55
*GAPDH*	TCTGTCGTGGACCTGACCTGC	GCCAGCACCCGCATCAAA	60
*IGF-1*	AGGTCGTCCATCGTAGTCCTTGCACTTTT	ACAGCGTCGTTATCGTTCCTGCAAACACAGGCCAAGGTAG	55
*IL-6*	TTCGACGAGGAGAAATGCTT	CCTTATCGTCGTTGCCAGAT	55
*TNF-α*	ATGAACCCTCCTCCGTACAC	AGAGGCCACCACATGATAGC	60

## Data Availability

The original contributions presented in this study are included in the article. Further inquiries can be directed to the corresponding author(s).

## Supplementary Materials

The following supporting information can be downloaded at https://www.mdpi.com/article/10.3390/vetsci12090861/s1; Figure S1: The original western blot pictures 1; Figure S2: The original western blot pictures 2.
