# Supplementary material for: Effects of Dietary Bacillus subtilis and Bacteriophage Supplementation on Water Quality, Carcass Traits, and Muscle Growth in Magang Geese
Source: Vet Sci. 2025 Sep 4;12(9):861. doi: 10.3390/vetsci12090861 (PMC12474057; doi:10.3390/vetsci12090861)

Figure S1

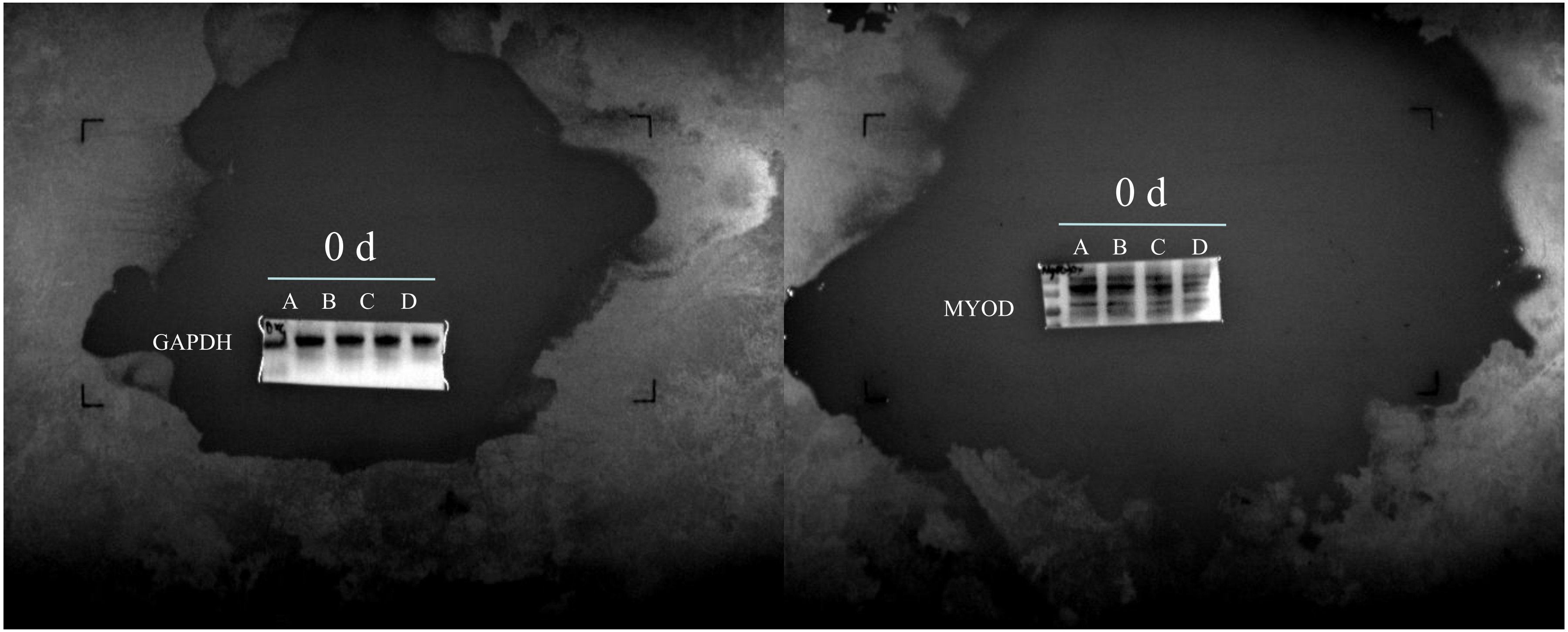

Figure S1

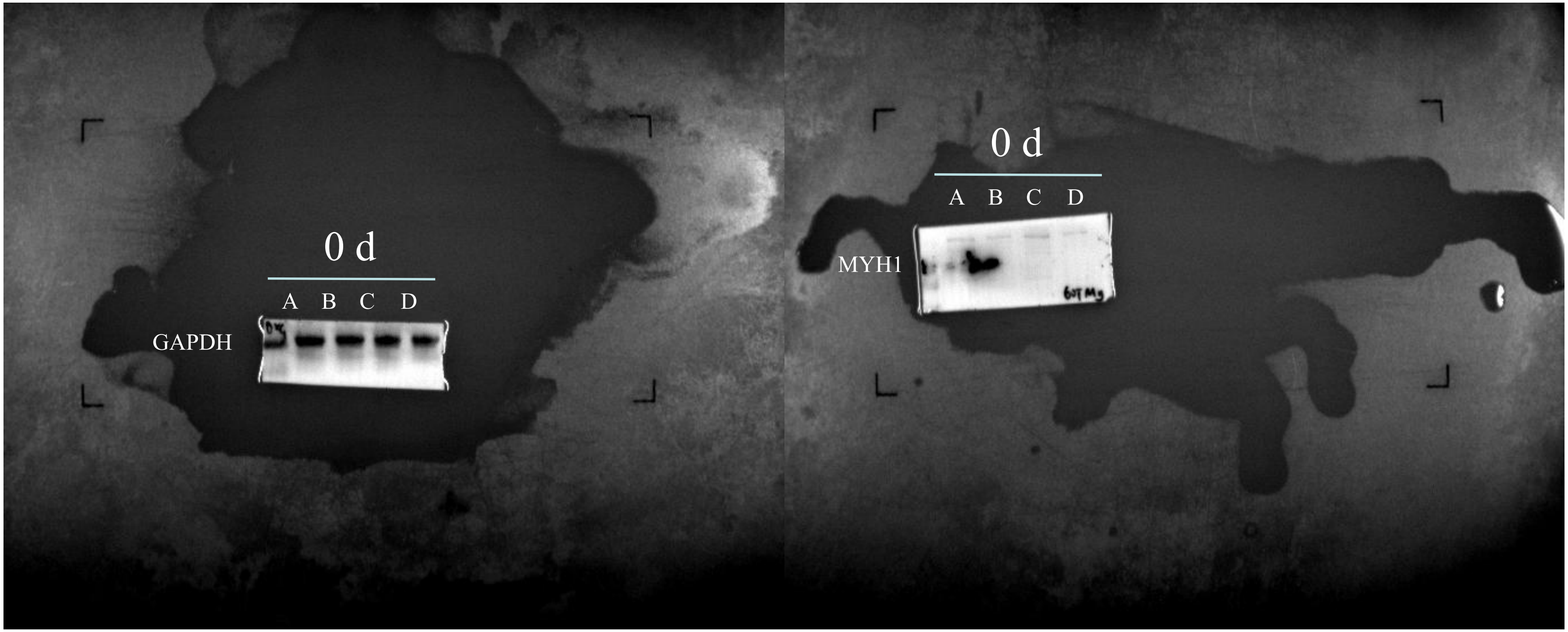

Figure S1

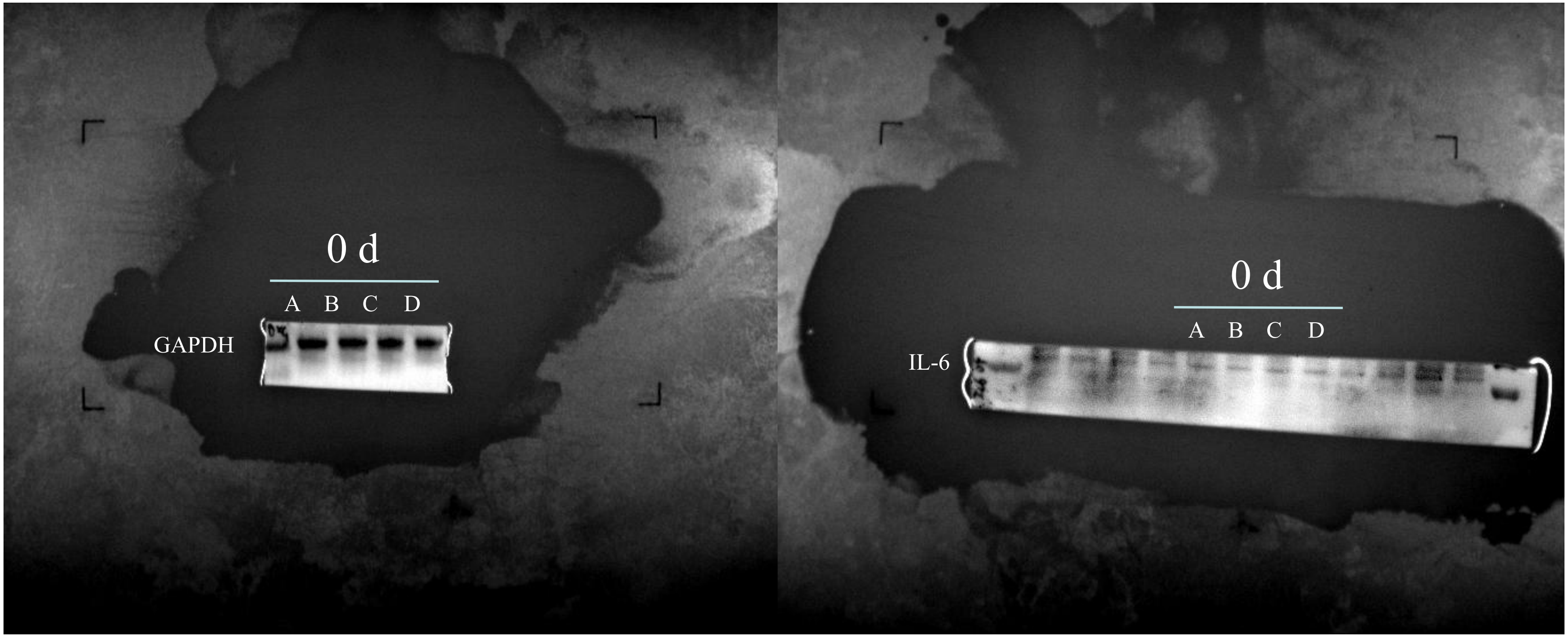

Figure S1

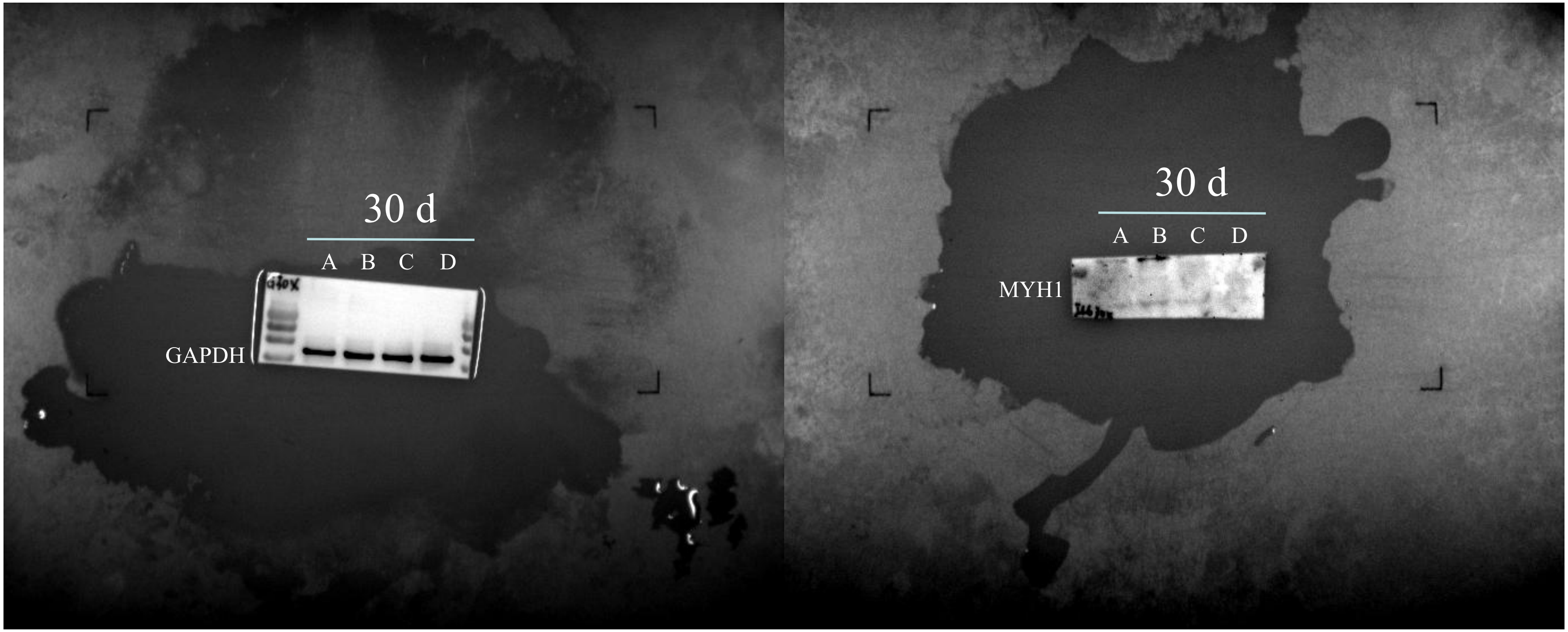

Figure S1

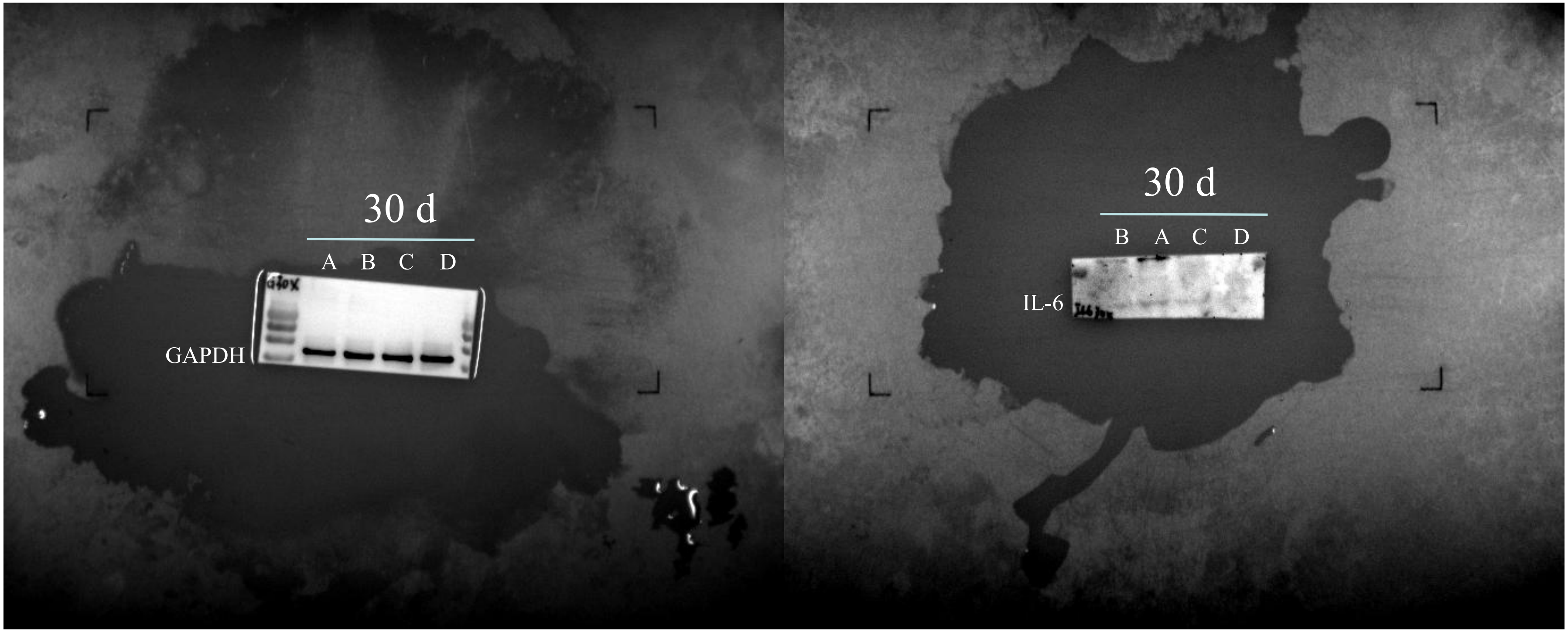

Figure S1

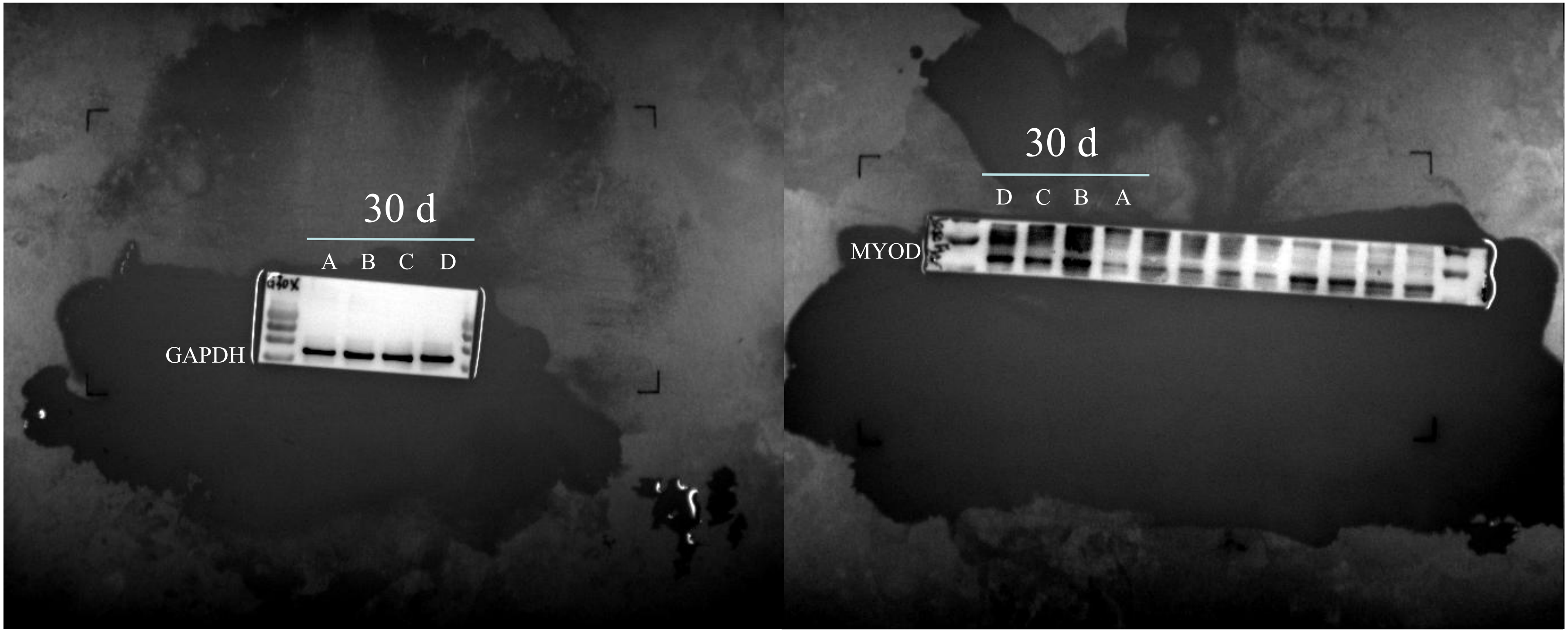

Figure S1

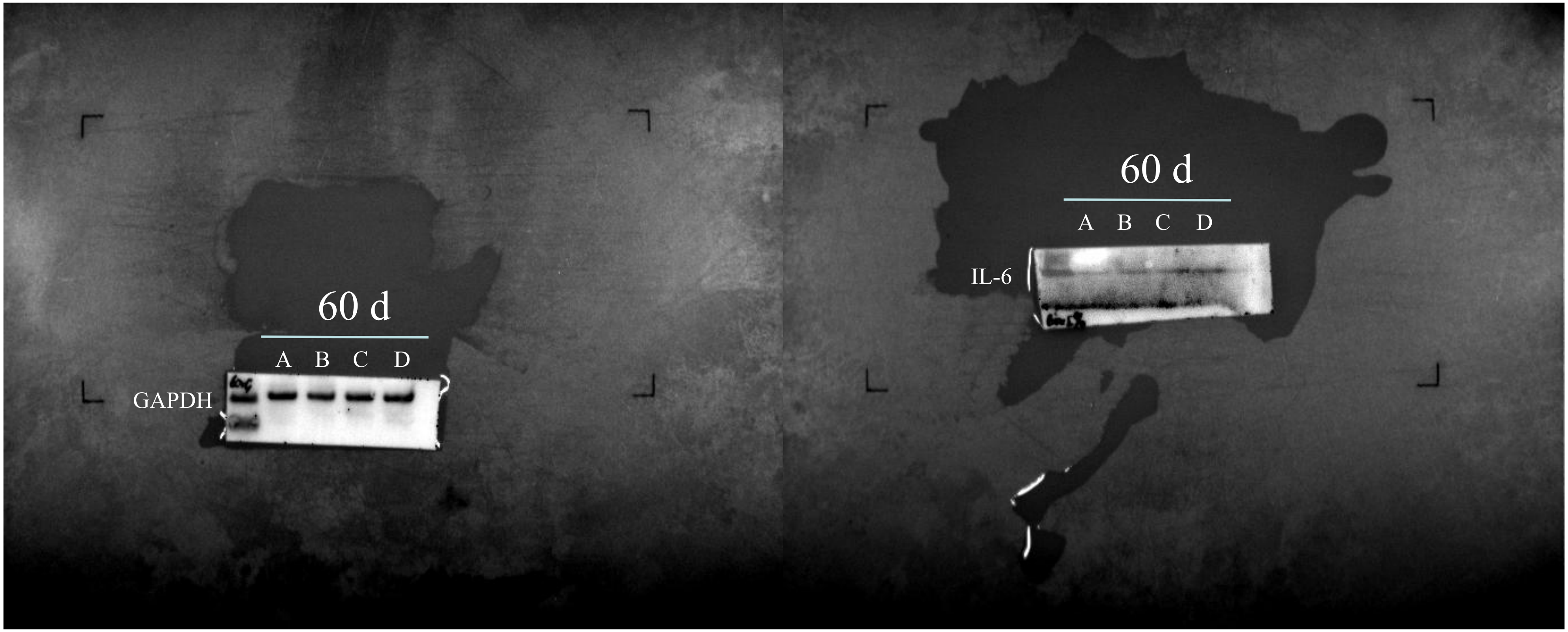

Figure S1

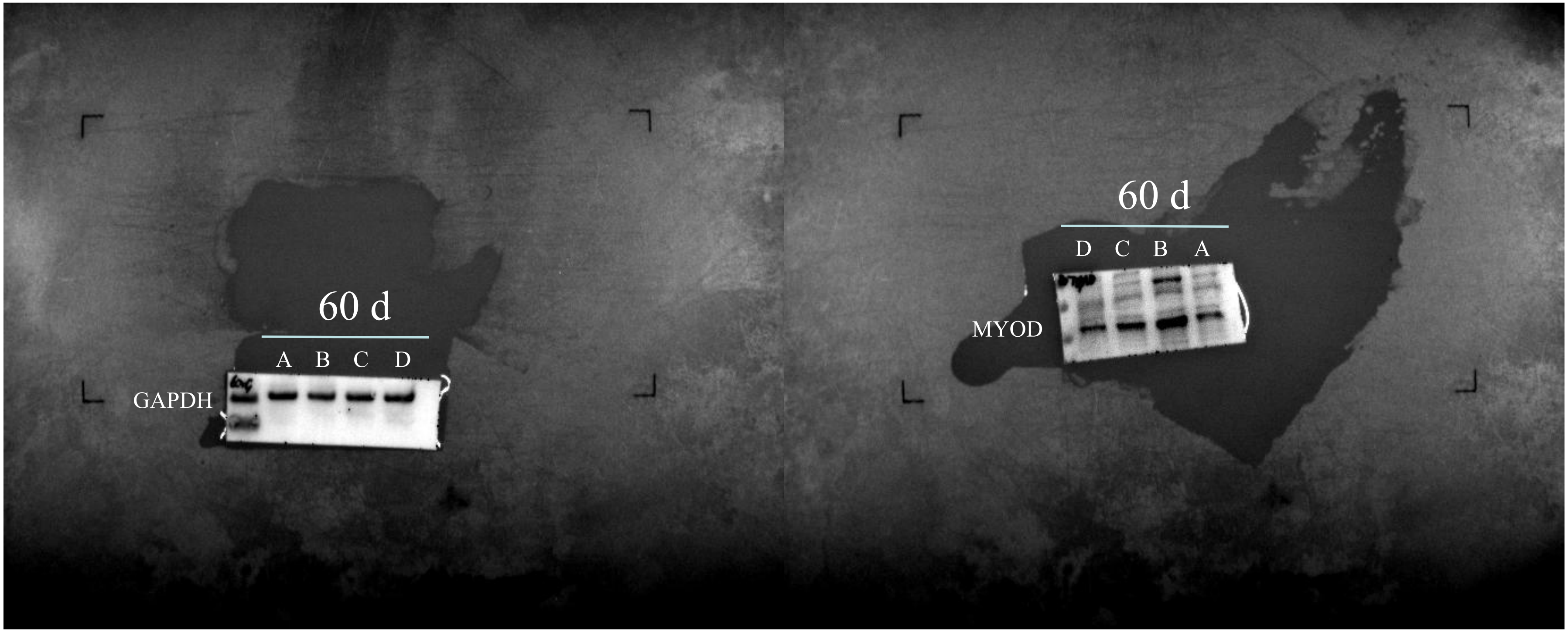

Figure S1

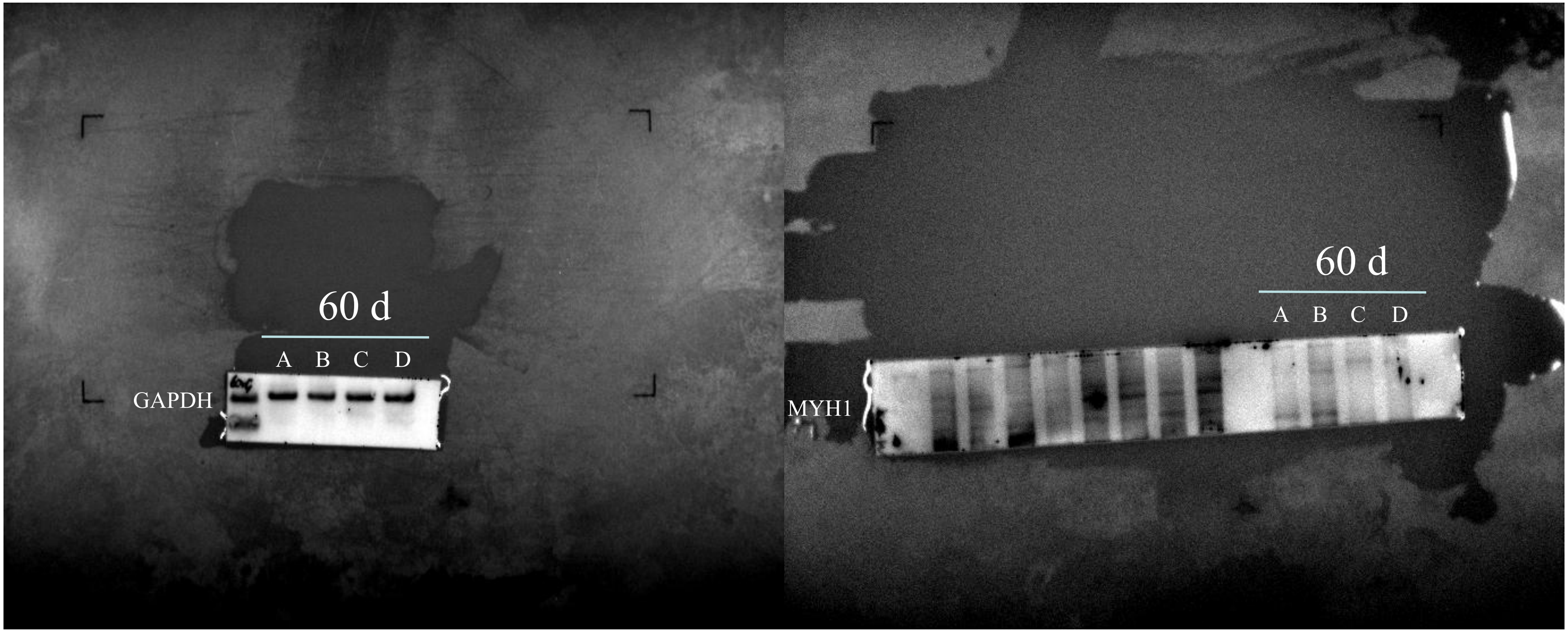

Figure S2

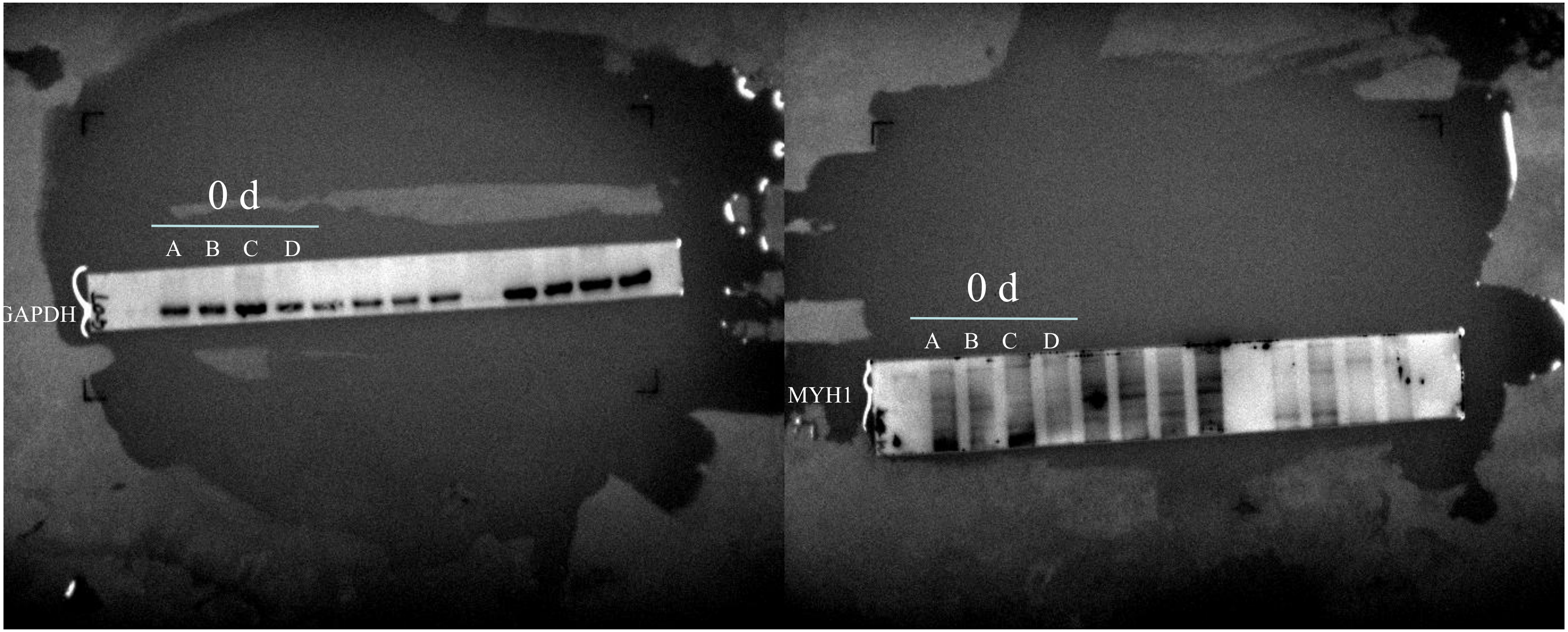

Figure S2

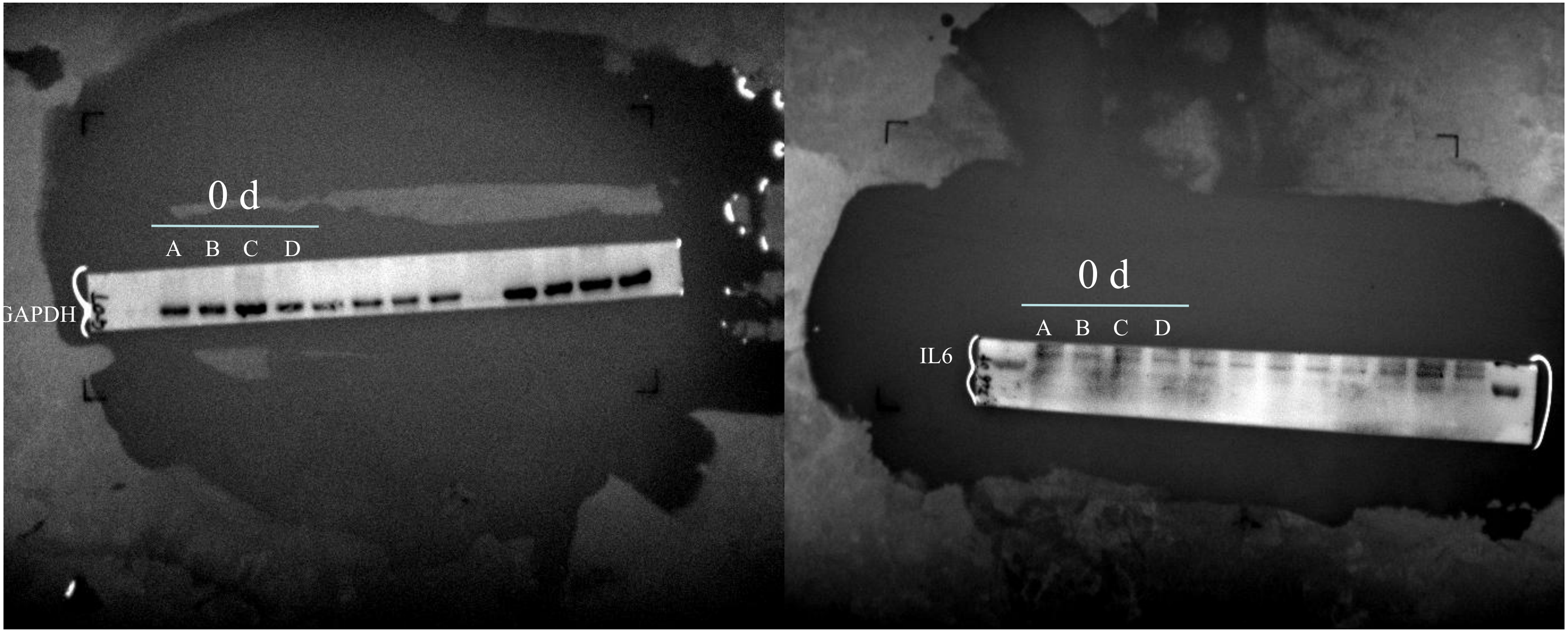

Figure S2

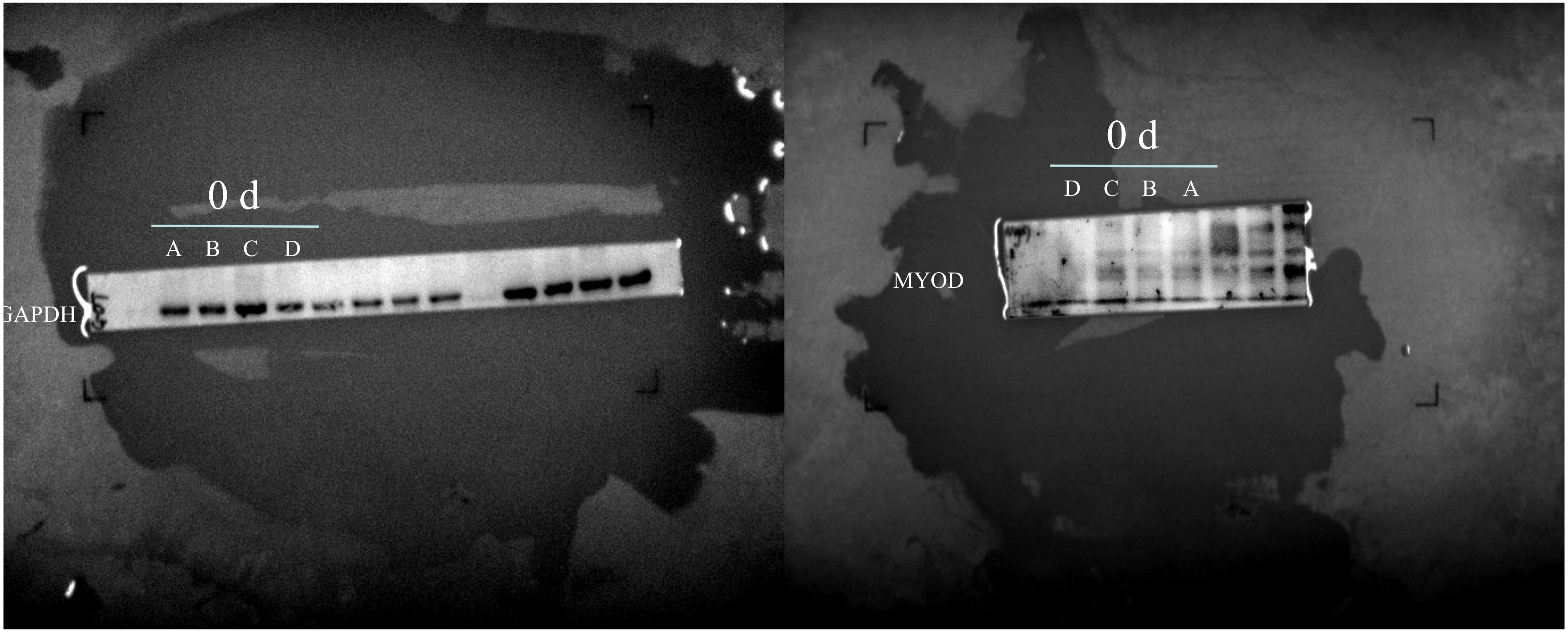

Figure S2

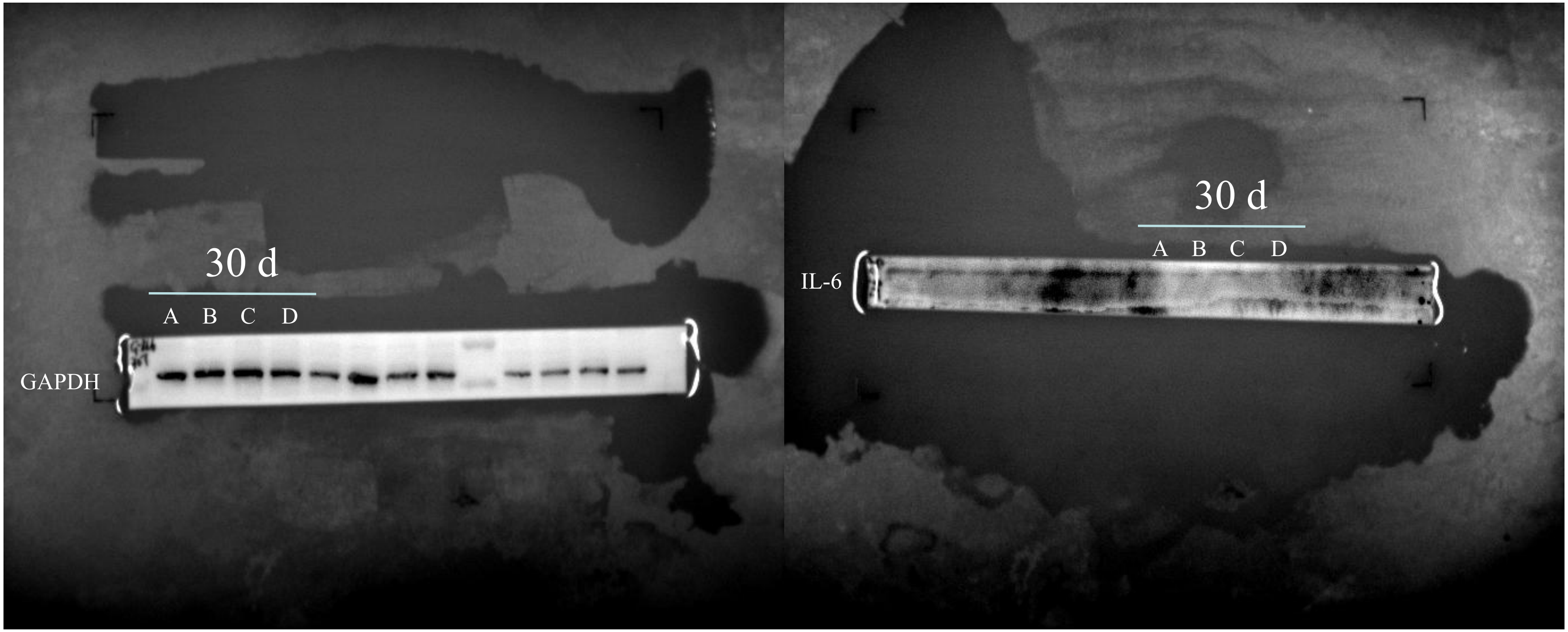

Figure S2

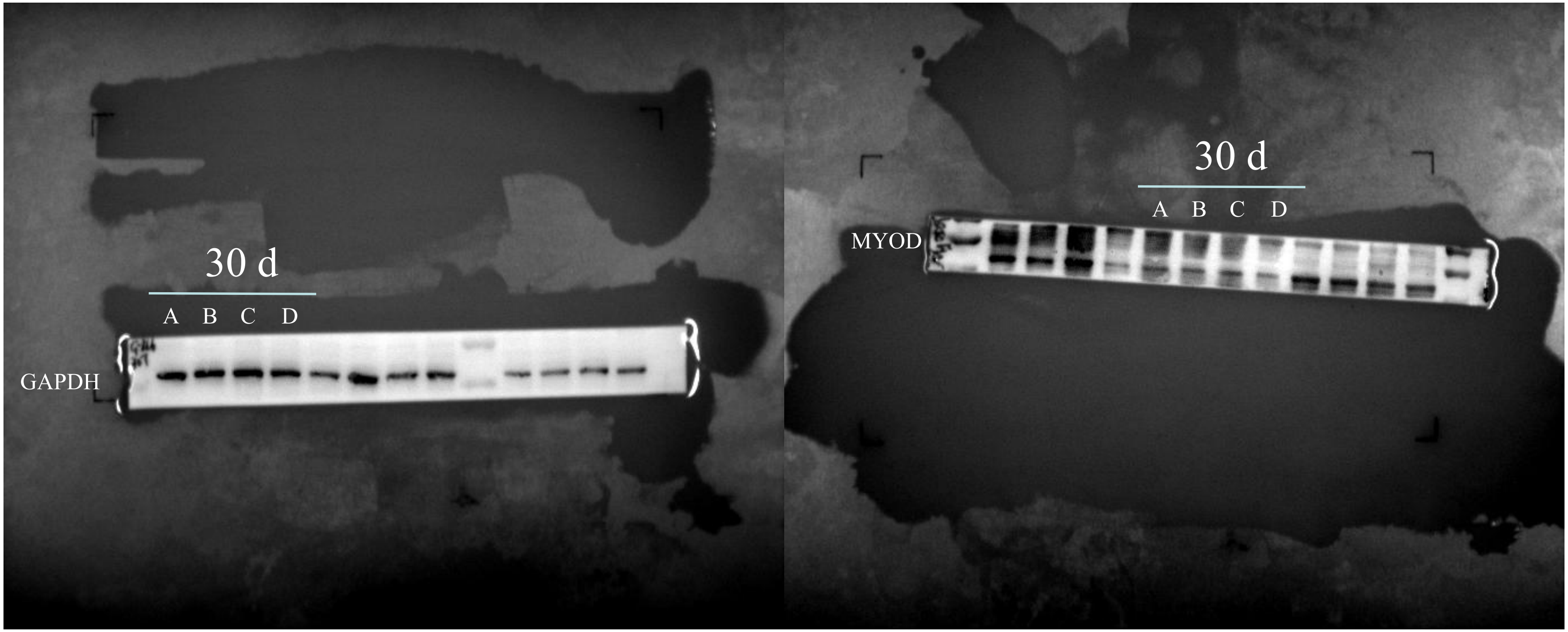

Figure S2

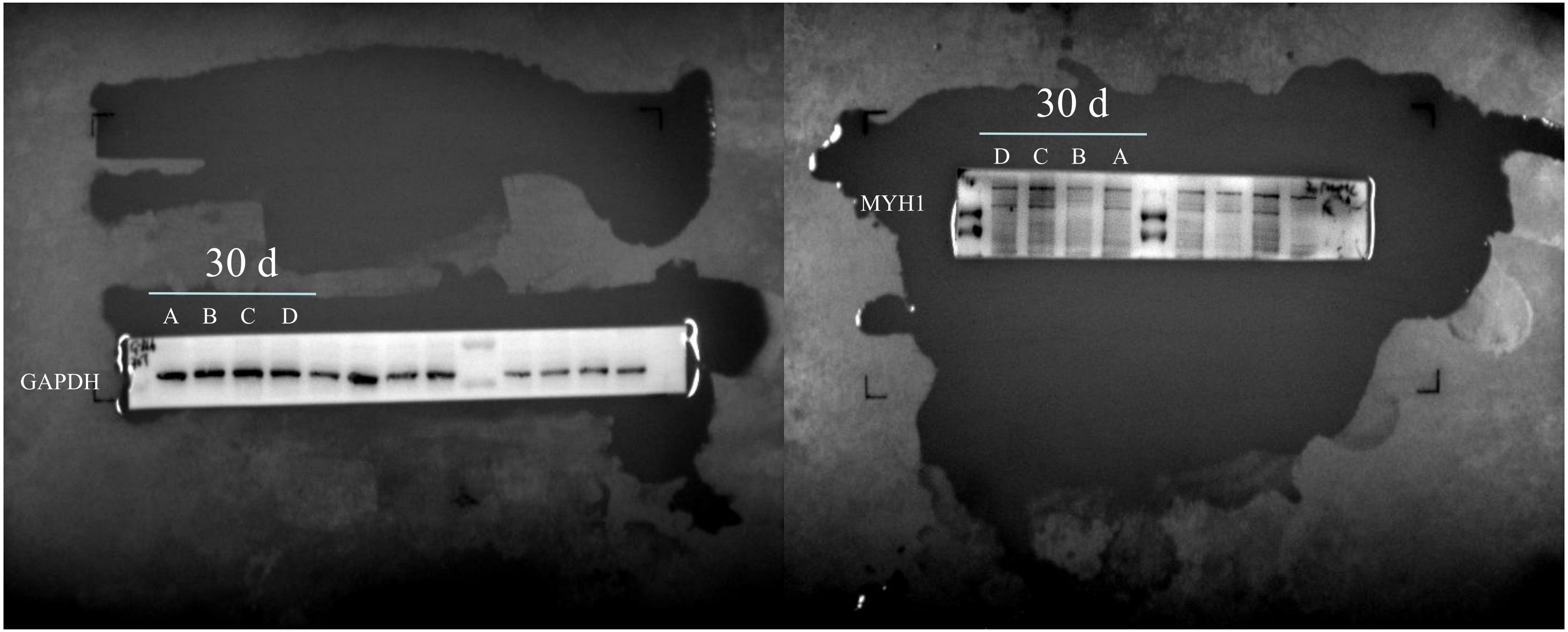

Figure S2

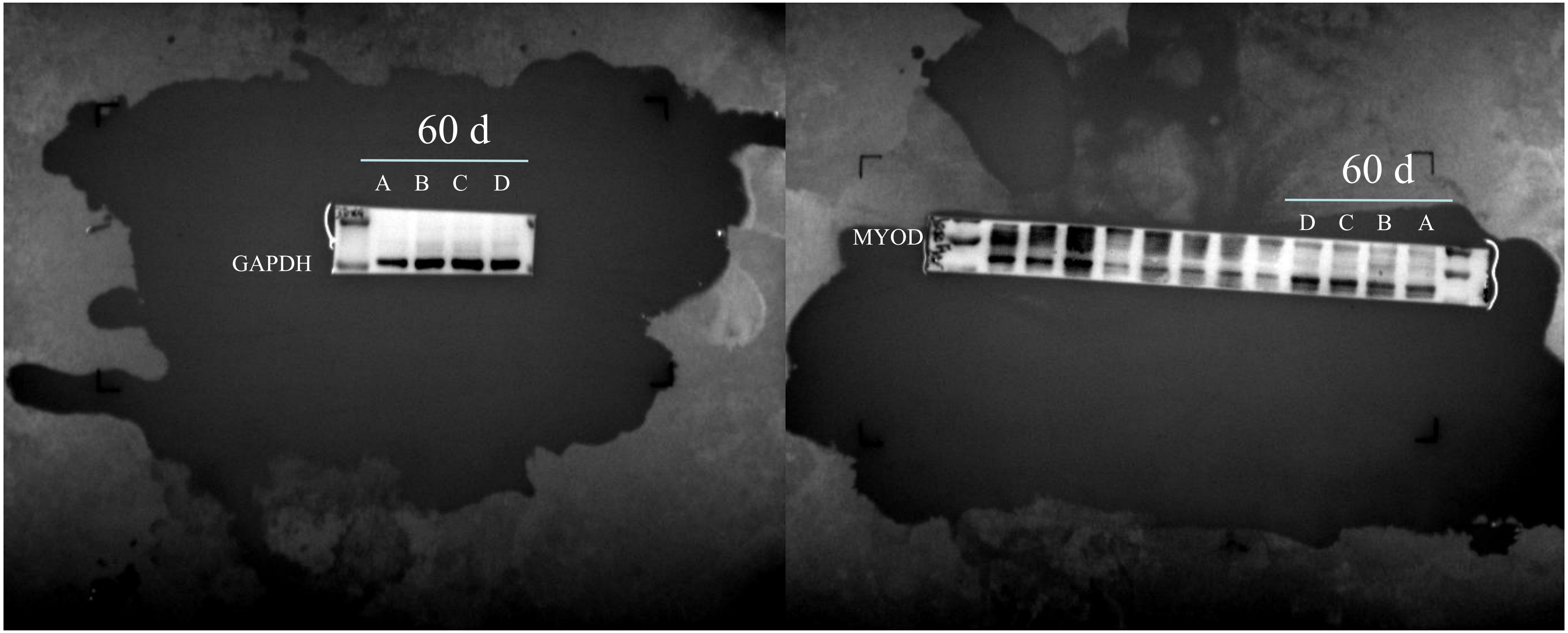

Figure S2

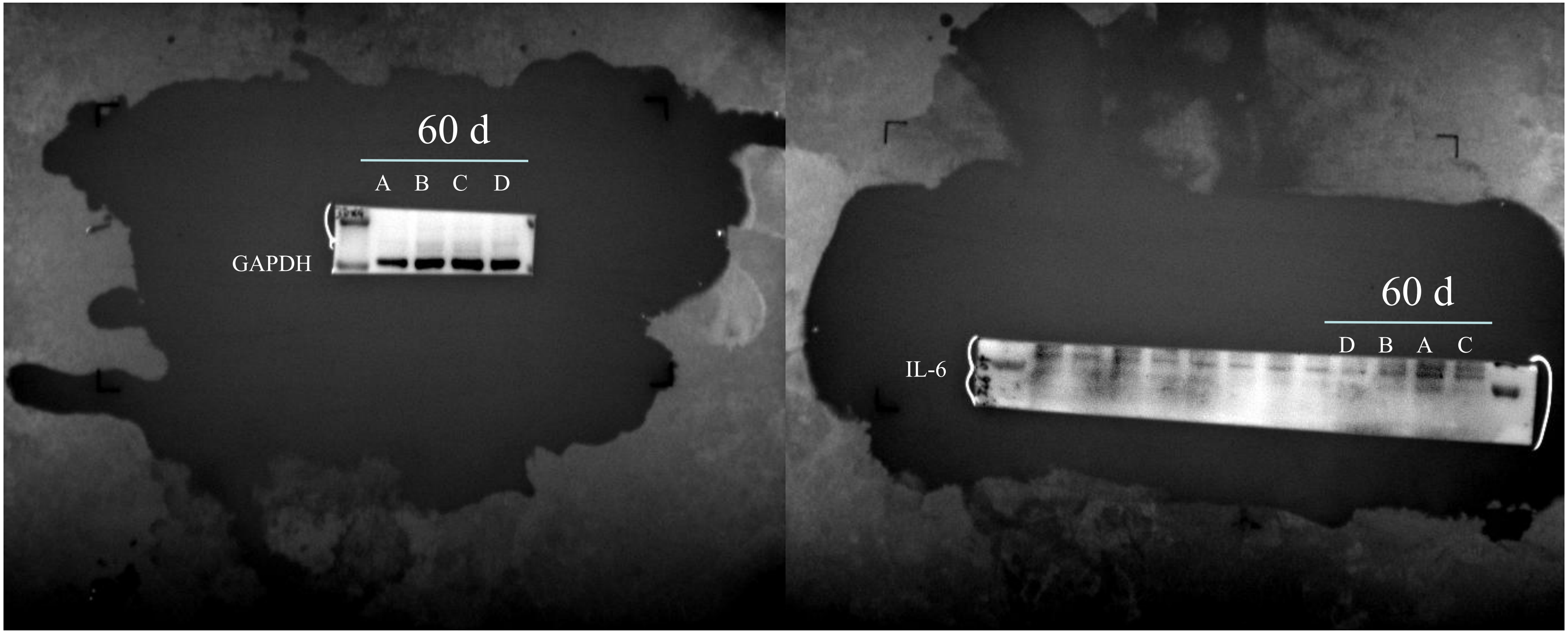

Figure S2

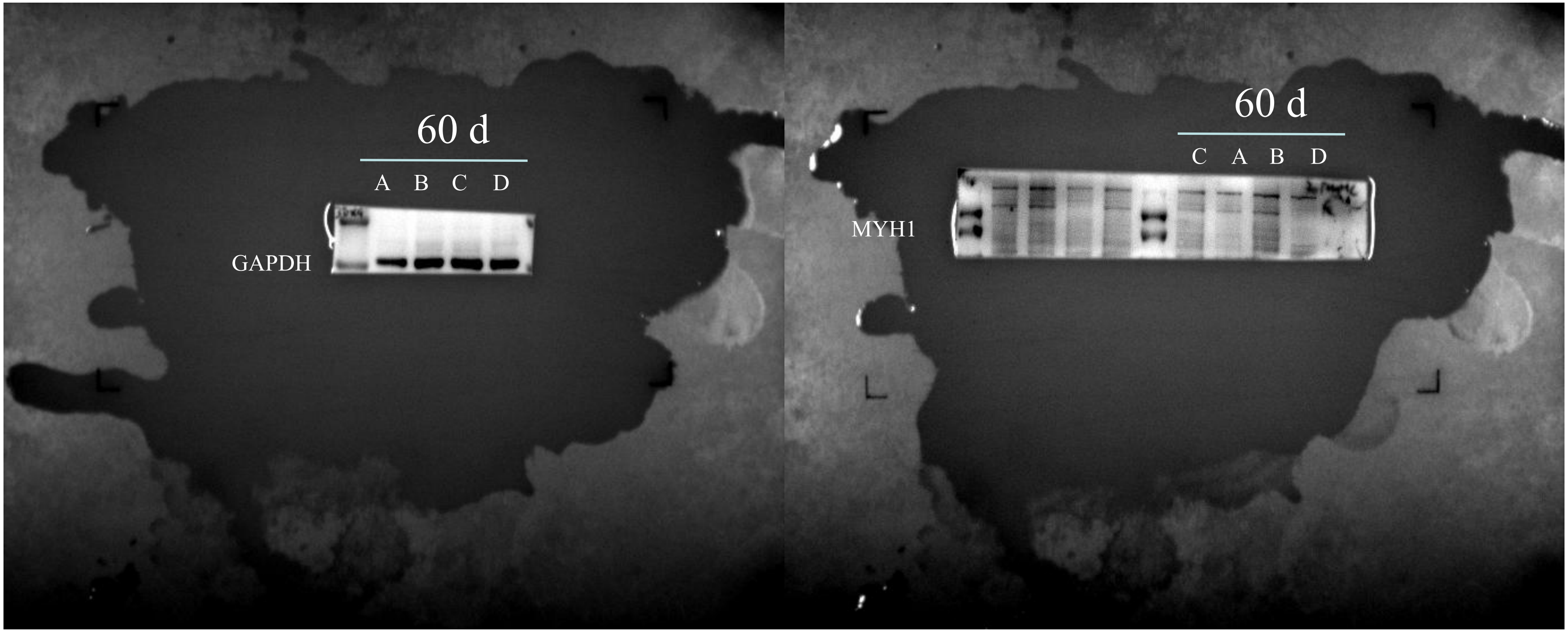

Supplement: Supplementary file 1 [file vetsci-12-00861-s001.zip › vetsci-3803631-supplementary.pdf]
